# Identification PMS1 and PMS2 as potential meiotic substrates of CDK2 activity

**DOI:** 10.1371/journal.pone.0283590

**Published:** 2023-03-23

**Authors:** Nathan Palmer, S. Zakiah A. Talib, Christine M. F. Goh, Kajal Biswas, Shyam K. Sharan, Philipp Kaldis

**Affiliations:** 1 Institute of Molecular and Cell Biology (IMCB), A*STAR (Agency for Science, Technology and Research), Singapore, Republic of Singapore; 2 Department of Chromosome Biology, Max Perutz Labs, University of Vienna, Vienna Biocenter, Vienna, Austria; 3 Department Biologie II, Biozentrum der LMU München, Zell- und Entwicklungsbiologie, Planegg-Martinsried, Germany; 4 Mouse Cancer Genetics Program, Center for Cancer Research, National Cancer Institute, National Institutes of Health, Frederick, MD, United States of America; 5 Department of Clinical Sciences, Clinical Research Centre (CRC), Lund University, Malmö, Sweden; 6 Lund University Diabetes Centre, Lund University, Clinical Research Centre (CRC), Malmö, Sweden; China University of Science and Technology, CHINA

## Abstract

Cyclin dependent-kinase 2 (CDK2) plays important functions during the mitotic cell cycle and also facilitates several key events during germ cell development. The majority of CDK2’s known meiotic functions occur during prophase of the first meiotic division. Here, CDK2 is involved in the regulation of meiotic transcription, the pairing of homologous chromosomes, and the maturation of meiotic crossover sites. Despite that some of the CDK2 substrates are known, few of them display functions in meiosis. Here, we investigate potential meiotic CDK2 substrates using *in silico* and *in vitro* approaches. We find that CDK2 phosphorylates PMS2 at Thr337, PMS1 at Thr331, and MLH1 *in vitro*. Phosphorylation of PMS2 affects its interaction with MLH1 to some degree. In testis extracts from mice lacking *Cdk2*, there are changes in expression of PMS2, MSH2, and HEI10, which may be reflective of the loss of CDK2 phosphorylation. Our work has uncovered a few CDK2 substrates with meiotic functions, which will have to be verified *in vivo*. A better understanding of the CDK2 substrates will help us to gain deeper insight into the functions of this universal kinase.

## Introduction

In meiosis, genetic information is exchanged between maternal and paternal chromosomes via meiotic recombination. This process is essential for the normal segregation of chromosomes at the first meiotic division [1] and also the generation of genetically heterogeneous gametes [2]. Meiotic recombination is initiated by programmed DNA double strand breaks induced by the topoisomerase-like transesterase, SPO11 [3]. Subsequent processing of meiotic DSBs leads to either crossover (CO) or non-crossover (NCO) products [4]. COs occur at only a minority of meiotic break sites with the vast majority being repaired as NCO. In mice, at least one or two COs usually form per homolog pair. In rare cases, three or more COs can form, which is more common in females than in males [5]. The majority of COs formed are also widely spaced and exert ‘‘interference” towards each other ensuring that no two COs occur too closely together [6]. The selection process whereby COs are formed instead of NCOs remains poorly understood. Currently, it is known that this selection involves the selective localisation of factors (ZMM proteins in budding yeast—Zip1-Zip2-Zip3-Zip4-Spo16, Msh4-Msh5, and Mer3 [7, 8]) to sites of meiotic recombination destined for CO formation. Orthologs of many of these ZMM proteins can be found in mice and humans and include RNF212 (Ring Finger Protein 212) [9, 10], CNTD1 (Cyclin N-Terminal Domain Containing 1) [11], HEI10 (Human enhancer of invasion 10) [10], EXO1 (Exonuclease 1) [12], and MER3 (ATP-dependent DNA helicase MER3 homolog) [13].

Our previous work indicated that cyclin-dependent kinase 2 (CDK2) also acts as a pro-crossover factor and is required for the selection process which designates specific sites of meiotic recombination to form meiotic COs [14]. There are two phosphorylation sites on CDK2 that affect its kinase activity. Phosphorylation of Tyr15 inhibits CDK2 kinase activity whereas Thr160 phosphorylation [15] is required for the full activation of CDK2/cyclin complexes. Increasing CDK2 kinase activity by preventing inhibitory phosphorylation, as in *Cdk2*^*Y15S*^, elevated the number of MLH1 (MutL protein homolog 1)-positive COs [16]. In contrast, replacing Thr160 by alanine effectively abrogated the late steps of CO designation by preventing COs to be formed between homologs [14]. Meiosis of *Cdk2*^*T160A*^ mutant spermatocytes was subsequently arrested during the transition of prophase I to metaphase I, preventing the first meiotic division and the formation of mature gametes required for fertility [14, 17].

DNA mismatch repair (MMR) complexes maintain genomic integrity by repairing mismatches generated during DNA replication and recombination. Recognition of mismatches by the MMR machinery can lead the excision of short or long patches of DNA and is followed by re-synthesis to restore the DNA sequence to that of a template DNA, often the sister chromatid. Such repair safeguards the genome from rearrangements that can result from nonallelic homologous recombination [18]. MMR is executed via the endonuclease activity of the MutL homolog protein, MLH1, in conjunction with one of two other human MutL homologues, PMS1 (Postmeiotic Segregation Increased 1) and MLH3 (MutL Homolog 3). The loss of MMR components is associated with both genomic instability [19, 20] and infertility [21–28], highlighting roles for both mitotic and meiotic divisions.

The association of MLH1 with PMS2, PMS1, or MLH3 results in the formation of the MutLα [29, 30], MutLβ, and MutLγ heterodimers, respectively [31, 32]. Although most eukaryotes have PMS1, MLH1 and MLH3, PMS2 has arisen more recently in the eukaryotic evolution [33, 34]. A role of MutLβ in MMR could not be detected in *in vitro* studies [35]. Nevertheless, MutLγ is able to repair base-base mismatches and small insertion-deletion loops, but its *in vivo* role may not be that important [36]. In addition, the PMS2 subunit is the active site of the MutLα endonuclease, which is conserved in MLH3 but not in PMS1 [37]. Based on this information, it is not surprising that MutLα and MutLγ are active in MMR but MutLβ is not.

In mice, the mismatch repair (MMR) proteins MSH2 and MSH3, which together form the MutSβ complex, localise to centromeric regions of the synaptonemal complex in addition to weaker interstitial binding sites and were observed to bind chromatin associated with these regions following ChIP-sequencing of pachytene stage spermatocytes [38]. Relevant to these findings was the fact that inappropriate localisation of both MLH1 and MLH3 to these sites was observed in the absence of PMS2, a component of the related MutLα complex (MLH1/PMS2). Here, significant accumulation of MLH3 was observed at centromeric and Y chromosome associated regions suggesting a redistribution of MLH-associated complexes at these repetitive sites. Interestingly, MLH3 foci at these sites were heterochromatic in their distribution and did not overlap with canonical DSBR proteins such as RAD51, suggesting this function was independent of DSB repair. The MSH2–MSH3 complex has been described to have an anti-recombinational role [39] and may prevent inappropriate MutLγ directed recombination in favour of MutLα directed MMR repair at these sites.

Unlike *Mlh1* and *Mlh3* deficient mice, only few *Pms2KO* mice produce spermatocytes proficient for CO formation [40], which can complete both meiotic divisions and generate spermatozoa [26]. Infertility in *Pms2KO* mice results from poorly described synapsis defects which lead to the loss of most spermatocytes before the completion of meiosis I and therefore a poor sperm count [26]. The endonuclease activity of PMS2 has since been found not to contribute towards fertility [41]. One potential explanation might be that repair of repetitive sequences are mediated by mismatch repair pathways independent of MutSγ and MutLγ which are reliant upon PMS2 –for example by PMS2 in association with MLH1 (as part of the MutLα heterodimer). The failure to repair such repetitive regions would then result in synapsis defects in areas which fail to properly complete repair. In a model where the activity of the MutLα heterodimer might be mediated via phosphorylation of the PMS2 subunit, we would expect that synapsis could be delayed or incomplete in the absence of the mediating kinase. Here we propose that CDK2 is expressed at the right time and in the right place to possibly phosphorylate PMS2. To date, the importance of MutLα during mammalian meiosis remains uncertain as is its role in mediating synapsis during meiotic prophase. Therefore, further study is needed to understand why the components of both MutL complexes (MutLγ–MLH1+ MLH3 and MutLα–MLH1 + PMS2) are needed during meiosis and why the phenotypes of *Pms2* deficient mice are different from *Mlh1* and *Mlh3* deficient mice.

Human enhancer of invasion 10 (Hei10) [42, 43] is a potential CDK2 substrate. The function of HEI10 in meiosis was originally identified in an infertile mouse mutant (*Mei4*) generated by *N*-ethyl-*N*-nitrosourea-induced mutagenesis [44]. Like *Cdk2*^*T160A*^ mutants, *Hei10* mutant or deletion models show normal homolog synapsis but defective localisation of MLH1/MLH3 to meiotic recombination sites [10, 44, 45]. The current hypothesis is that HEI10 functions during zygonema by limiting the colocalization of RNF212 with MutSγ-associated recombination sites resulting in the early differentiation between CO and NCO sites [9, 10]. At a later stage, HEI10 is aided by MutLγ to accumulate at the designated CO sites. MLH1/MLH3 in addition to EXO1 [12, 46] is then presumed to execute the final steps of crossing over. RNF212 can be observed as many foci during the early stages of meiotic recombination and foci are reduced in number until mid-pachytene when RNF212 can only be observed at late recombination nodules [9, 10]. After deletion of *Hei10*, a high number of RNF212 foci can be detected despite normal synapsis of homologs–which is similar in our *Cdk2*^*T160A*^ model [10, 14]. Therefore it is possible that CDK2 and HEI10 function in the same pathway or depend on each other to promote the selection of RNF212 marked sites which will later develop into late recombination nodules. As alterations to RNF212 and HEI10 dynamics are a common theme in mutants lacking proteins which localise to late recombination nodules, we considered HEI10 as a potential CDK2 substrate.

The functions of CDK2 have been extensively studied in the mitotic cell cycle and these studies have revealed that there are hundreds of substrates that are phosphorylated by CDK2 [47–49]. These studies were done either in yeast or in cell lines and among the identified substrates there are few with known functions in meiosis. Since the identification of meiotic substrates of CDK2 is challenging for technical reasons, we used a candidate approach to identify potential substrates of CDK2 *in silico* and tested them using *in vitro* kinase assays. We identified PMS2 and PMS1 as CDK2 substrates. Phosphorylation of T337 in PMS2 affected the interaction with MLH1 but its endonuclease activity was not affected. In addition, we tested additional substrates for CDK2 *in vitro* but could only find a few that were phosphorylated by CDK2.

## Materials and methods

### Mutant mouse lines used in this study

*Cdk2*^*T160A*^ [17] and *Cdk2KO* [50] mice have been reported and were maintained on a C57BL/6 background. Mice were housed under standard conditions, maintained on a 12-hour light/dark cycle, fed a standard chow diet containing 6% crude fat, and were treated in compliance with the institutional guidelines for animal care and use. Mice were anaesthetized with avertin or ketamine and were euthanized using carbon dioxide. All animal experiments were done at and experimental protocols were approved by the Animal Care and Use Committee of Biological Resource Centre at Biopolis, A*STAR, Singapore (protocol#171268).

### Recombinant protein expression and purification

All recombinant protein expression was performed in Dh5α^™^ *E*. *Coli* cells (Invitrogen; cat#18265017) as previously reported [51] and bacteria were lysed in a French press at 18,000 lb/in^2^ followed by ultracentrifugation at 25,000 rpm for 45 min at 4°C. Purified recombinant proteins were stored in 50 mM HEPES (pH 8.0), 10% glycerol, 150 mM NaCl, 1 mM DTT, 10ug/ml pepstatin, 10mg/ml leupeptin and 10mg/ml chymostatin at -80°C.

### Kinase assays

Kinase assays were done as previously described [52] using 1μg of recombinant protein, which was incubated with 100 ng of active CDK2/cyclin A2 complexes, 30μM ATP (Roche; 10127523001), and 5 μCi of [gamma-^32^P] ATP (PerkinElmer; NEG502A) in EB buffer (EBN without NP-40) for 30 min at room temperature. The levels of incorporated radioactivity were quantified with a PhosphoImager (Fujifilm; FLA-7000).

### Protein extraction and Western blotting from cells or tissues

Protein were extracted with RIPA buffer or EBN buffer for western blots, immunoprecipitations, and kinase assays as has been described in detail [53].

### Endonuclease activity measurements

*Cdk2*^*ko/ko*^;*p53*^*ko/ko*^ [54];*p53*^*ko/ko*^ [54], and *Pms2*^*ko/ko*^ [55] MEFs have been described previously. Cell lines were grown in DMEM (Gibco) +10% FBS (Hyclone) + 1X Penicillin-Streptomycin-Glutamine (Gibco) at 37°C with 5% CO_2_ and 3% O_2_ supplemented incubators. The endonuclease assays was done as has been described in detail previously [55, 56].

### Antibodies

The following antibodies were used in this study: MLH1 [BD Bioscience; cat#BD554073 (1/1000)], PMS2 [BD Bioscience; cat#BD556415 (1/1000)], HSP90 [BD Bioscience, cat#BD610419 (1/5000)], GAPDH [Cell Signaling, cat#5174 (1/1000)], MSH2 [BD Bioscience, cat#BD556349 (1/1000)], CDK2 [Santa Cruz; cat#sc-6248(1/400)], HEI10 [Abcam; cat#Ab71977 (1/500)], MYC-tag [Cell Signaling Technology; cat#2276 (1/2000)], V5-tag [Cell Signaling, cat#13202 (1/1000)], MLH1 [BD Bioscience; cat#BD554073 (1/1000)], and gamma-tubulin [Sigma-Aldrich; cat#T6557 (1/5000)].

### Statistical analysis

The experiments were repeated at least three times. Quantitative data (mean ± s.e.m.) were analyzed using Student’s t-test in GraphPad Prism version 6 (GraphPad Software, La Jolla, CA, USA) and differences were considered statistically significant when p<0.05.

## Results

### *In silico* identification of meiotic substrates of CDK2/cyclin A2

Since few CDK2 substrates with known functions in meiosis have been identified previously, we checked the amino acids sequence of meiotic proteins for CDK2 phosphorylation sites (S/TPxK/R; [57]) considering also data from the PhosphoSitePlus (www.phosphosite.org) website. Among the proteins that we considered as potential substrates, were PMS1, PMS2, MLH1, BLM, BRCA2, EXO1, RAD1, HEI10, and MER3. It turns out that all of these proteins have Ser/Thr residues that could potentially be phosphorylated by CDK2.

**Fig 1:**
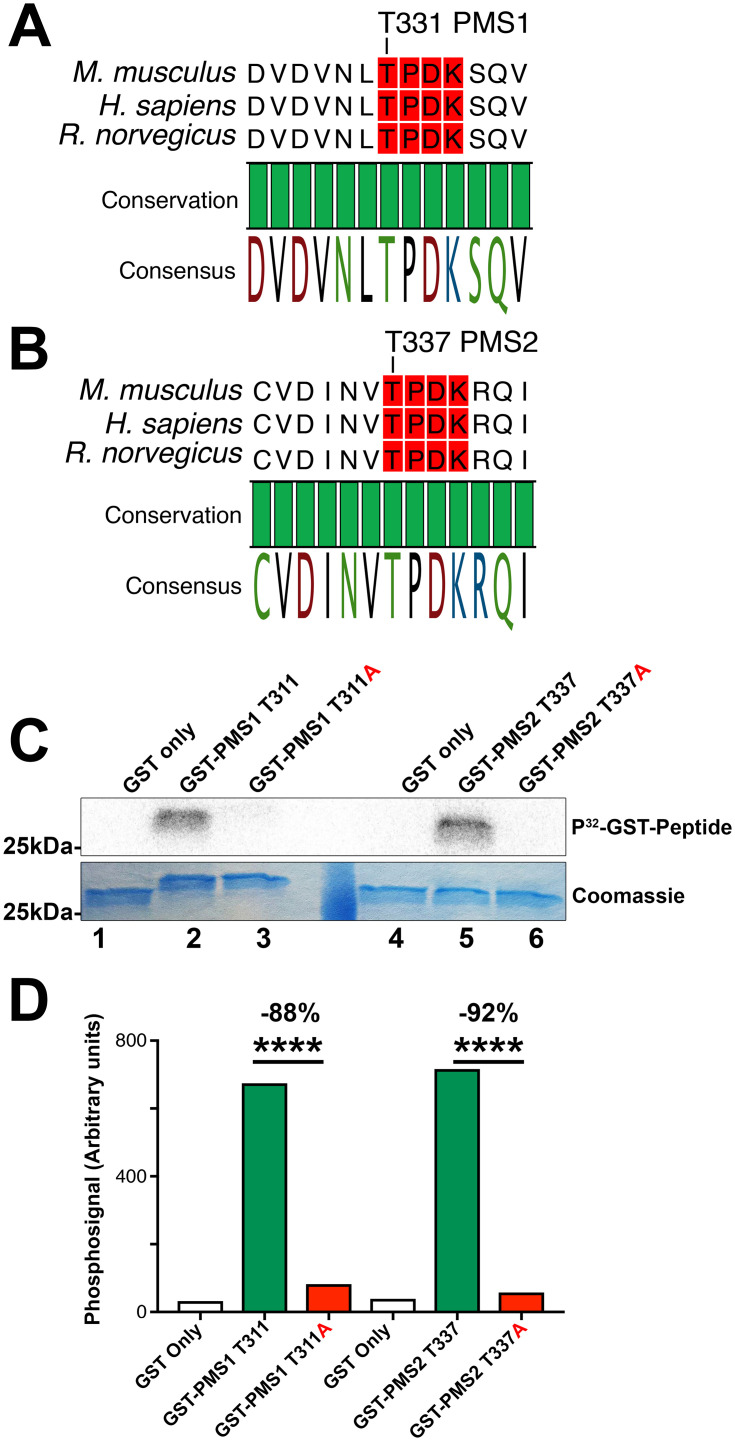
Potential CDK2 substrates; PMS1 and PMS2. Graphic representation of the evolutionary conservation of Thr331 and Thr337 in PMS1 (A) and PMS2 (B), respectively. Amino acid positions are labelled relative to the transcripts from *Mus musculus*. The CDK consensus site in each sequence is highlighted in red. (C) CDK2/cyclin A2 kinase assays directed against GST-tagged fusion peptides of PMS1 Thr331 (lane 2) or PMS2 Thr337 (lane 5) or their threonine-alanine point mutants (lanes 3 and 6, respectively). GST tag without fusion peptide are shown in lanes 1 and 4 as negative controls. Upper panel shows the phospho-image of each peptide and represents the amount of radiolabelling occurring following phosphorylation by CDK2/cyclin A2. The lower panel shows the Coomassie staining of radiolabelled proteins to indicate equal loading in each lane. (D) Phospho-image quantification of peptides from panel A. Data is presented as the absolute level of phosphorylation observed in each peptide as determined by densitometry analysis. Significance is determined by the difference in quantified phospho-signal by unpaired-T test. This experiment was repeated at least three times with similar results observed in each case.

In terms of PMS1 and PMS2, the MMR activity of MutLα [58] and the stability of this complex [59] have previously been reported to be sensitive to phosphorylation but the kinases that phosphorylate these proteins are not known. We uncovered that Thr331 in PMS1 (Fig 1A) and Thr337 in PMS2 (Fig 1B) might be targets of CDK-mediated phosphorylation since these residues fit the criteria of an ideal CDK consensus site for phosphorylation [57]. In addition, the lysine at the +4 position (K334/340) of the putative CDK consensus site belongs to the ATP binding site of PMS1/2.

### Investigation of PMS2 Thr337 as a substrate of CDK2/cyclin A2

In order to investigate experimentally whether Thr337 in PMS2 or Thr331 in PMS1 are indeed substrates of CDK2, we performed *in vitro* kinase assays using purified proteins. We cloned 15 amino acid peptides corresponding to Thr337 of PMS2 and the related Thr311 of PMS1 in fusion with glutathione S-transferase (GST), expressed it in *E*. *coli*, and purified these proteins. Using these purified substrates, we observed phosphorylation of both PMS1 and PMS2 mediated by CDK2/cyclin A2 (Fig 1C). Phosphorylation was specific to these sites as mutagenesis to non-phosphorylatable alanine (T337A or T331A, respectively) was sufficient to ablate the observed phosphosignal under the same conditions (Fig 1C, lanes 3 and 6, respectively), while GST alone (containing 14 S/T residues) was not phosphorylated (Fig 1C, lanes 1&4). Quantification of the PhosphoImager data indicated a reduction of approximately 90% when Thr was replaced by Ala (Fig 1D). Based on these experiments, we conclude that CDK2 can phosphorylate T331 in PMS1 and T337 in PMS2 *in vitro*.

**Fig 2:**
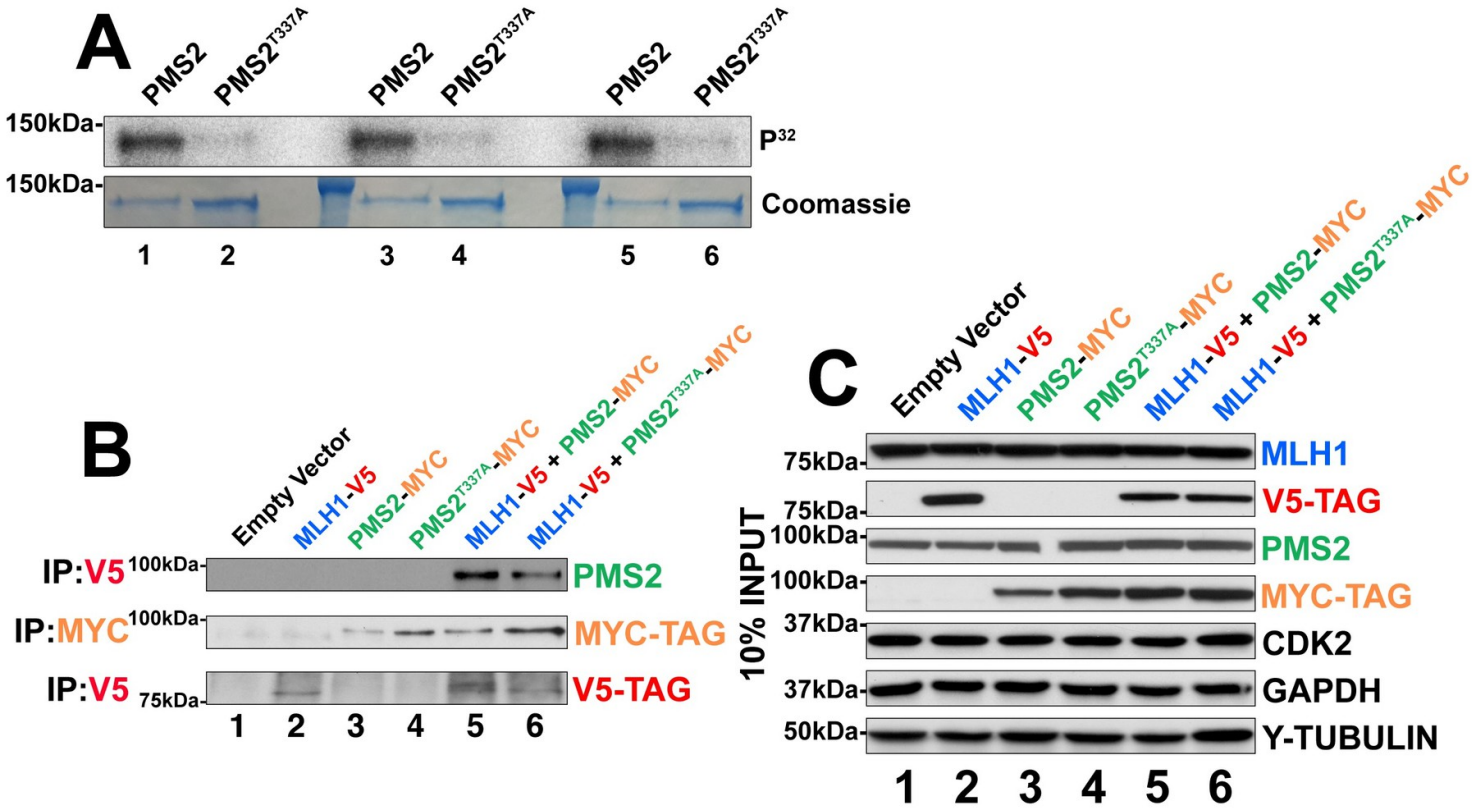
CDK2 phosphorylation of full-length PMS2 and its effect on the complex with MLH1. (A) CDK2/cyclin A2 kinase assay directed against GST-tagged fusion PMS2 (lanes 1,3,5) or PMS2^T337A^ (lanes 2,4,6) full length proteins. Upper panel shows the phospho-image of each protein and represents the amount of radiolabelling occurring following phosphorylation by CDK2/cyclin A2. The lower panel shows the Coomassie staining of radiolabelled proteins to indicate equal loading in each lane. (B) IP-western blot analysis of transfected MLH1-V5 or PMS2-MYC proteins from HEK293T cells. Antibodies used for immunoprecipitation are shown at the left hand side whilst antibodies used for detection are shown on the right hand side of the blot. Correct expression of tagged proteins is detected by the V5 or Myc tag following their respective immunoprecipitation (2^nd^ and 3^th^ panels from top, respectively). Co-immunoprecipitation of MLH1-V5 with PMS2-myc or PMS2^T337A^-myc is shown in lanes 5 and 6. (C) Input western blot of lysates used for panel A. Here 10% of the total protein used for immunoprecipitation in panel A was probed for proteins relevant for the assay. CDK2, GAPDH and gamma-TUBULIN are shown as loading controls. Molecular weight markers in kDa are indicated on the left.

In order to determine whether this was the only site on PMS2 phosphorylated by CDK2, we cloned full-length PMS2 downstream of a GST tag, purified the full-length protein, and performed kinase assays. Full-length PMS2 was phosphorylated readily by CDK2 in the *in vitro* kinase assays (Fig 2A, lanes 1, 3, 5). Mutagenesis of Thr337 to a non-phosphorylatable alanine in full length PMS2 reduced the observed signal by 92% (Fig 2A, lanes 2, 4, 6). These experiments indicated that Thr337 is most likely the major CDK2 phosphorylation site in PMS2 *in vitro*.

To determine whether phosphorylation of Thr337 might be important for the stability of MLH1-PMS2 complexes, we overexpressed both Myc-tagged PMS2 and V5-tagged MLH1 or PMS2^T337A^ together in HEK293T cells and determined the extent of interaction using immunoprecipitation. PMS2 and MLH1 could be readily detected in a complex (Fig 2B, top panel, lane 5). The interaction between V5-MLH1 and Myc-PMS2 was slightly decreased upon mutagenesis of Thr337. This was judged by the amount of PMS2 precipitated using antibodies against the V5-Tag on MLH1 (lowest panel of the IP western blot in Fig 2B; for input see Fig 2C) and quantification suggested a decrease of 46% of PMS2^T337A^ bound to MLH1 (nevertheless, this number should be taken with a grain of salt since western blot signals are not linear). Since these constructs were expressed in HEK293T cells which express many CDKs, we cannot specifically conclude that this result was due to the loss of CDK2-mediated phosphorylation as there may be other kinases in HEK293T cells that could phosphorylate PMS2 at T337. Nevertheless, these results indicate that post-translational modification of Thr337 in PMS2 slightly affects the stability of the MLH1/PMS2 complex in cells.

### Testis extracts from *Cdk2* mutant mice

**Fig 3:**
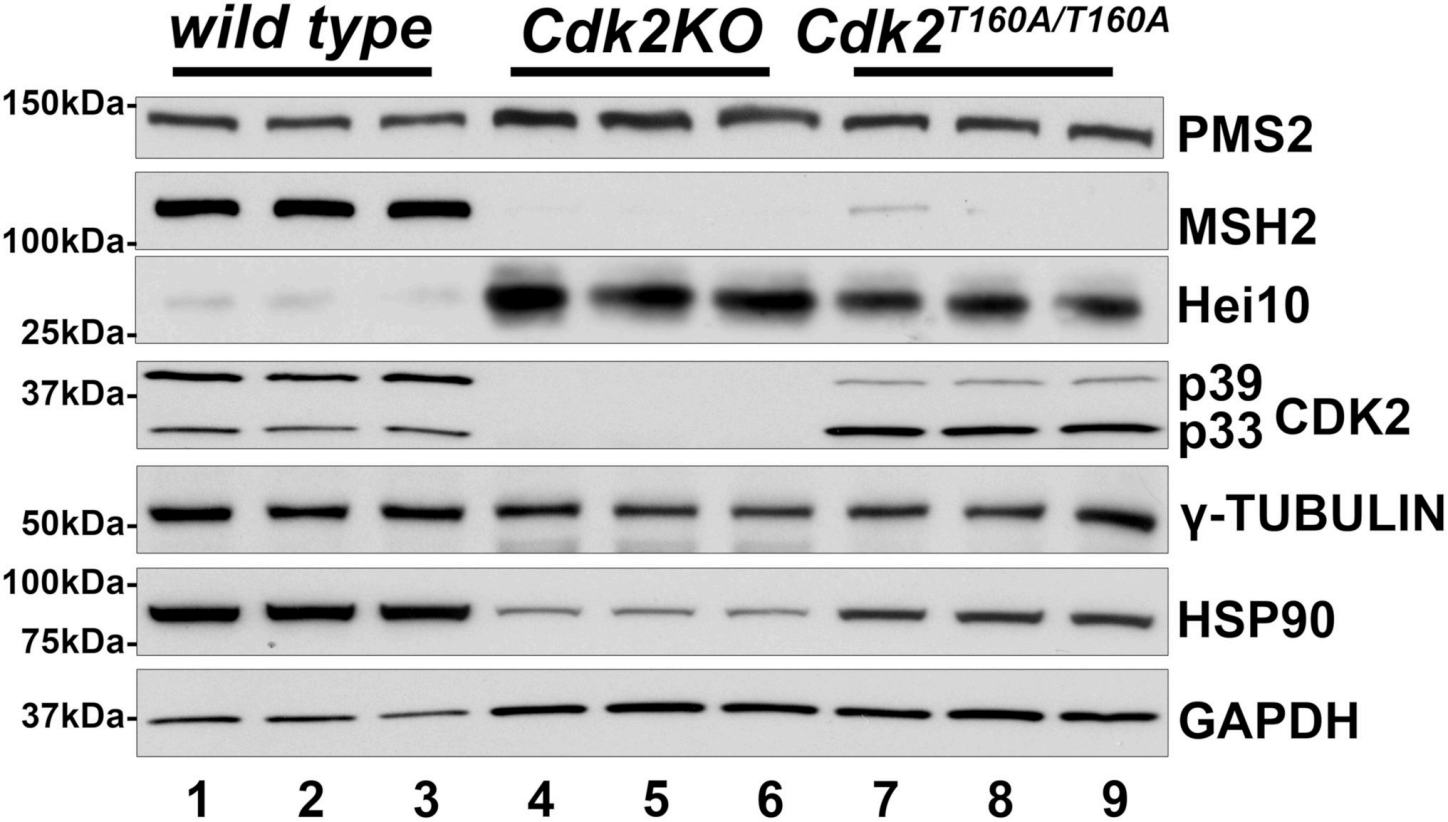
Protein analysis in WT, *Cdk2KO*, and *Cdk2*^*T160A*^ testis. Western blot analysis of testis lysates extracted from p56 wild type (lanes 1–3), *Cdk2KO* (lanes 4–6) or *Cdk2*^*T160A*^ (lanes 7–9) mice. Different biological replicates are used for each lane shown. gamma-TUBULIN, GAPDH, and HSP90 are shown to indicate equal loading of each lane. Molecular weight markers in kDa are shown on the left.

Our results suggest that CDK2/cyclin A2 is able to phosphorylate proteins with important meiotic functions *in vitro* including PMS2 and PMS1 (see Figs 1 and 2). In order to investigate our findings using alternative approaches, we used western blotting of testis extracts from WT, *Cdk2KO*, and *Cdk2*^*T160A*^ mice. We reasoned that in the absence of the CDK2 protein (*Cdk2KO*) or CDK2 activity (*Cdk2*^*T160A*^), T337 in PMS2 would not be phosphorylated (along with all other CDK2 substrates). This assumption is of course based on the hypothesis that CDK2 is the only kinase that phosphorylates T337 in PMS, which may not be true. As a readout, we investigated the effects on PMS2 protein expression as well as other mismatch repair proteins. We confirmed that CDK2 was expressed in WT but not in *Cdk2KO* testis (Fig 3, fourth panel from top, compare lanes 1–3 with 4–6). In addition, we found that CDK2^T160A^ was expressed in *Cdk2*^*T160A*^ testis at similar levels as in WT but there is a difference in the proportion of the long form of CDK2 (p39) versus the short form (p33) (Fig 3, fourth panel from top, compare lanes 7–9 to 1–3). Furthermore, we used three different control proteins (gamma-tubulin, GAPDH, and HSP90) to ensure equal loading of all lanes (Fig 3, bottom three panels; please note HSP90 is highly expressed in primary spermatocytes [60] which are depleted in *Cdk2KO* testis). Although PMS2 was readily detected in all genotypes at the correct molecular weight, a slightly increase in expression could be observed in *Cdk2KO* and *Cdk2*^*T160A*^ (Fig 3, top panel). It is important to note here that the stability of PMS2 might simply be increased by the fact that in *Cdk2KO* and *Cdk2*^*T160A*^ animals, meiosis never progresses to the stage where MLH1 foci can be observed. Prior experiments in non-meiotic cell lines have suggested that PMS2 is efficiently expressed only in the presence of MLH1 [61–63]. Additionally, cell lines lacking MLH1 expression are practically devoid of PMS2 protein, although normal *Pms2* mRNA levels are produced [61] (this is further discussed in the Discussion section). Unfortunately, MLH1 could not be detected in any of our lysates despite that this antibody works well for detecting CO foci in chromosome spreads [14]. Instead we used antibodies against MSH2, which together with MSH3 forms the MutSβ complex [64, 65]. MSH2 was well expressed in WT testis but we were not able to detect it in *Cdk2KO* and *Cdk2*^*T160A*^ extracts (Fig 3, second panel from top, compare lanes 1–3 to 4–9). In conclusion, we found that there were changes in protein expression in PMS2 and MSH2 in the absence of the CDK2 protein or activity but we cannot exclude that this is an indirect effect and may have little to do with Thr337 phosphorylation in PMS2.

### Endonuclease activity of PMS2

**Fig 4:**
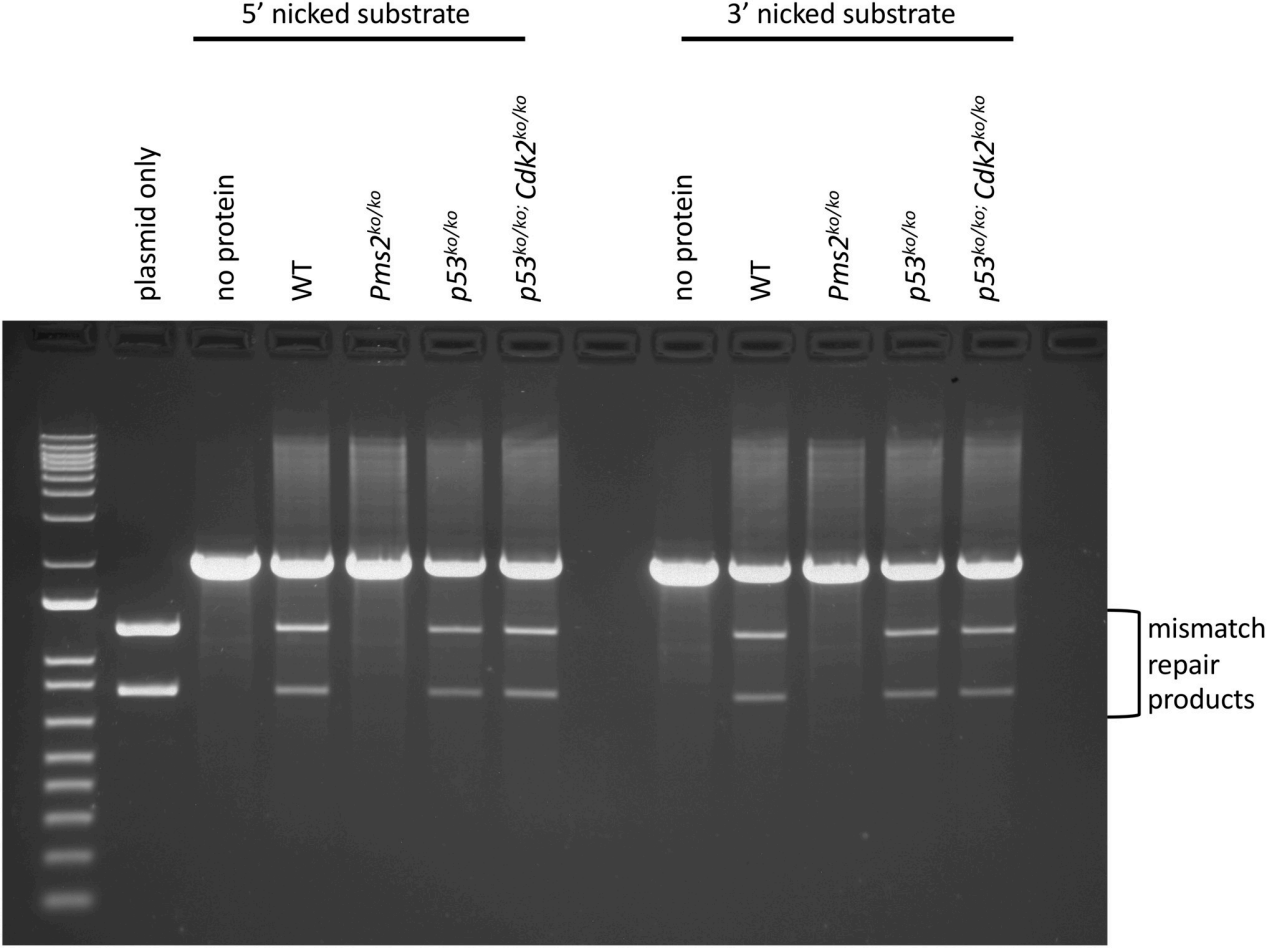
Endonuclease activity of PMS2. The endonuclease activity of PMS was measured *in vitro* using nuclear extracts from *p53*^*ko/ko*^;*Cdk2*^*ko/ko*^, *p53*^*ko/ko*^, *Pms2*^*ko/ko*^, and wild type (WT) MEFs. Heteroduplexes with either a 5’ or a 3’ nick were digested with AseI and PstI after incubation with nuclear extracts. Products of the digestions were run on agarose gels. The endonuclease activity of PMS2 leads to repair at the PstI site resulting in a 0.8kb and 1.2kb fragment. The far left lane shows a molecular weight marker. Controls included “plasmid only” and “no protein”.

In order to investigate the functional consequences of PMS2 phosphorylation by CDK2, we aimed to measure the endonuclease activity of PMS2. This assay includes incubation of nicked heteroduplex plasmids with nuclear extracts and running digestion products on agarose gels as described previously [55, 56]. For this experiment, we prepared nuclear extract from *Cdk2*^*ko/ko*^ MEFs that had been immortalized by knocking out p53 (*Cdk2*^*ko/ko*^*;p53*^*ko/ko*^). In order to control for the loss of p53, we also included *p53*^*ko/ko*^ MEFs (wild type for *Cdk2*). Additional controls included *Pms2*^*ko/ko*^ and wild type MEFs. WT extract containing normal PMS2 activity was able to repair both 5’ and 3’ nicks and displayed the mismatch repair products that would be expected (Fig 4). This has been described carefully in Figure 4D of [55]. In contrast, *Pms2*^*ko/ko*^ MEFs displayed no endonuclease activity (Fig 4), which serves as a negative control and confirms recent results [55]. To our surprise, both *Cdk2*^*ko/ko*^;*p53*^*ko/ko*^ and *p53*^*ko/ko*^ MEFs displayed a similar activity as wild type, indicating that the PMS2 endonuclease activity was not affected by the absence of CDK2 or p53. This result suggests that even if CDK2 phosphorylates PMS2, it is not likely to affect its endonuclease activity. Loss of p53 also does not affect the endonuclease activity, which is somewhat surprising since p53 plays major roles in DNA damage repair and the loss of p53 leads to increases in the activity of several CDKs [54]. At the same time, there are possible limitations of this experiment including (i) that PMS2 maybe phosphorylated by other CDKs since we have shown substantial overlap between CDKs [66–68] and (ii) nuclear extracts from MEFs have been used due to the requirement of large amounts of protein instead of meiotic extracts. In summary, we were not able to detect a defect in PMS2 activity in the absence of CDK2 but this will need to be further investigated in the future.

### MLH1 as a potential CDK2 substrate

**Fig 5:**
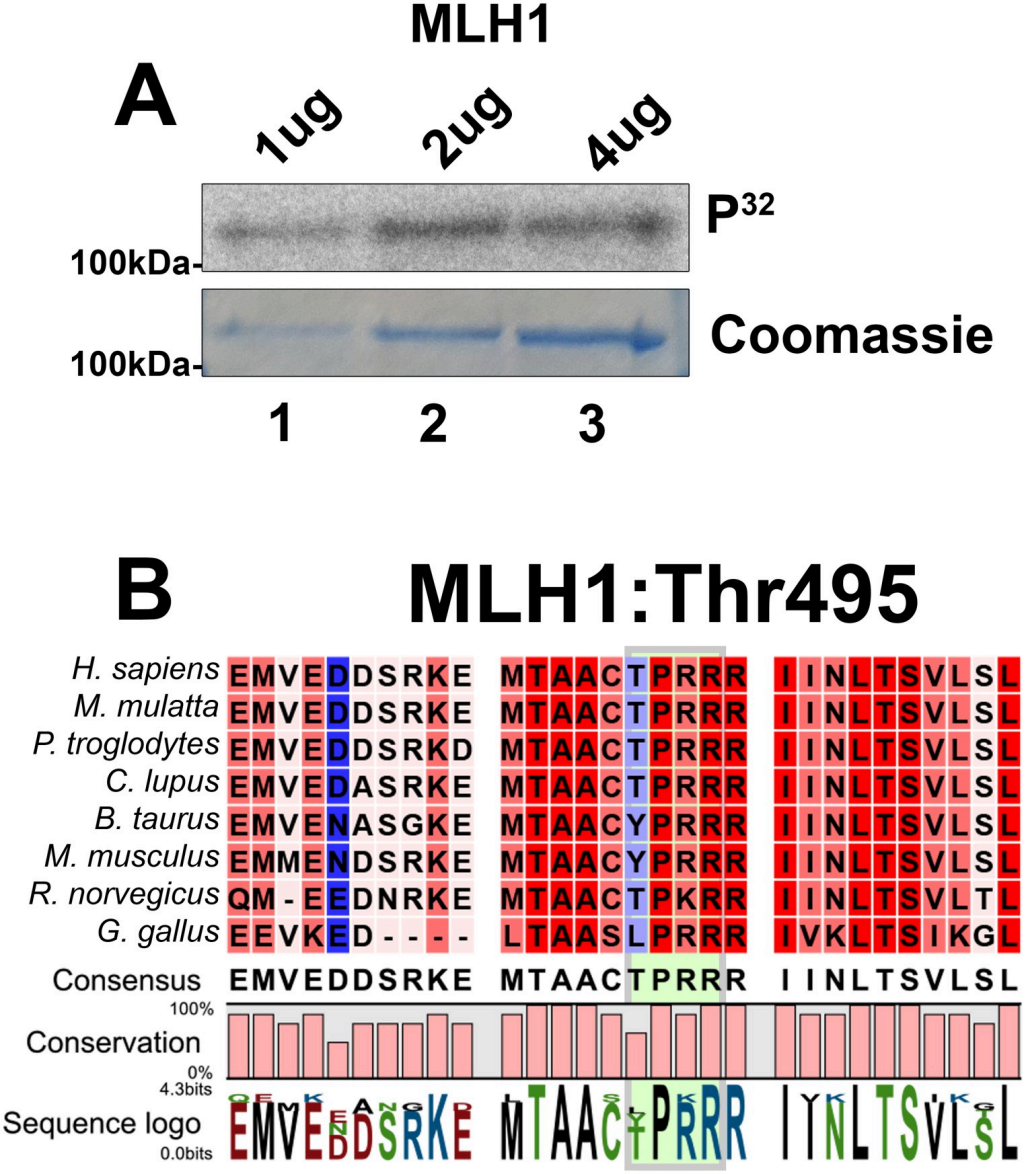
CDK2 phosphorylates full-length MLH1. (A) CDK2/cyclin A2 kinase assay directed against GST-tagged fusion MLH1 (all lanes). Increasing amounts of GST-MLH1 are used in lanes 1–3 as indicated. Upper panel shows the phospho-image of MLH1 and represents the amount of radiolabelling occurring following phosphorylation by CDK2/cyclin A2. The lower panel shows the Coomassie staining of radiolabelled proteins to indicate equal loading in each lane. (B) Sequence alignment of the evolutionary conservation of Thr495 in human MLH1. Amino acid positions are labelled relative to the transcripts from *H*. *sapiens*. The CDK consensus site is highlighted using the green box but is not conserved in the *M*. *musculus*, *B*. *taurus*, and *G*. *gallus* sequences.

Since PMS2 is unlikely to be the only CDK2 substrate, we also tested whether MLH1 was able to be phosphorylated by CDK2/cyclin A. We detected incorporation of radioactive phosphate into MLH1 (Fig 5A) most likely at Thr495, which is part of the TPRRR motif resembling a consensus CDK site [57] for phosphorylation (Fig 5B). Previous studies have suggested that MLH1 is unlikely to be functionally altered by phosphorylation [69]. This data indicates that MLH1 is a potential *in vitro* substrate of CDK2 although we were not able to determine the exact site that was modified.

### HEI10 is unlikely to be phosphorylated by CDK2/cyclin A

**Fig 6:**
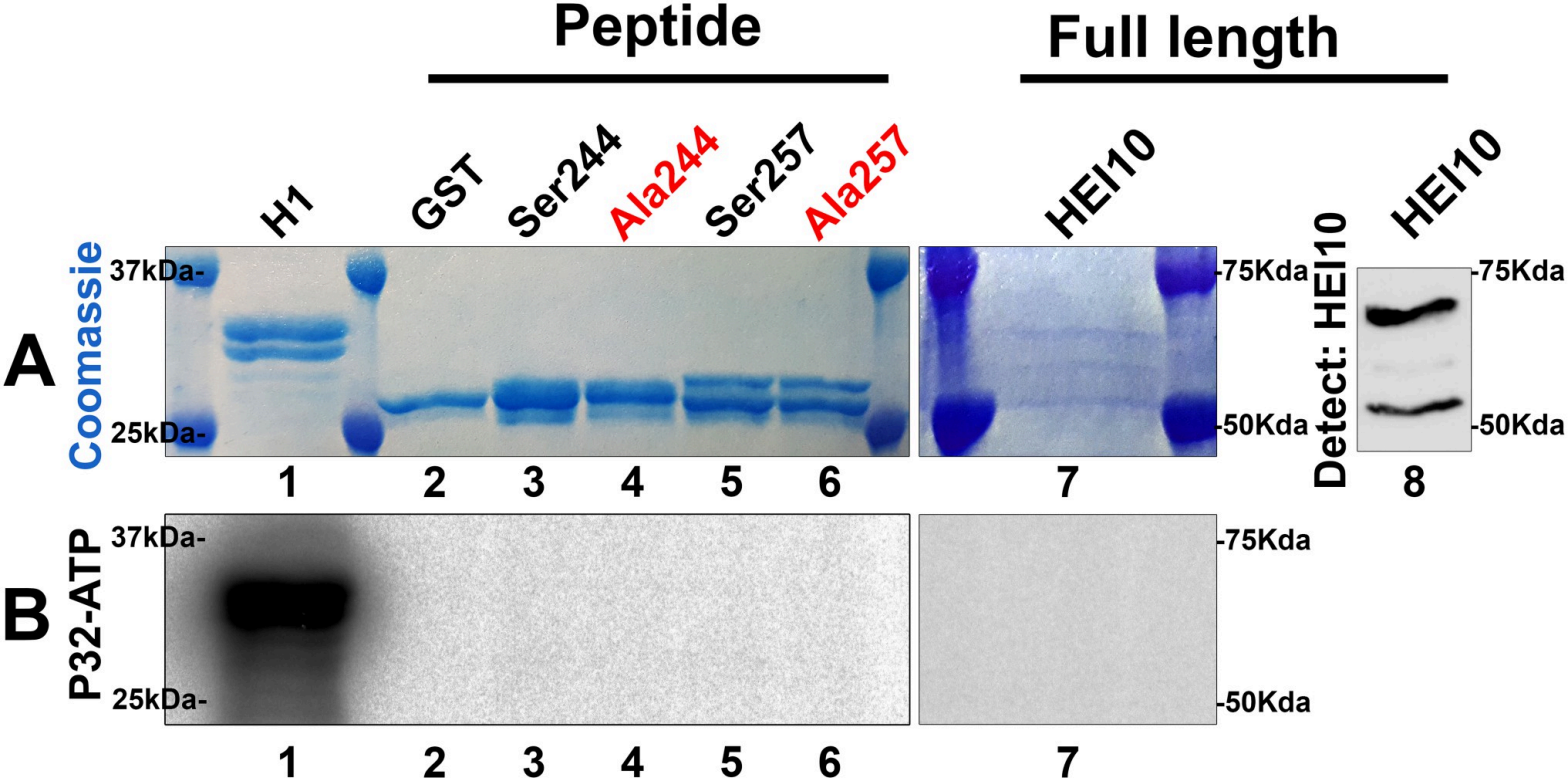
HEI10 as a potential CDK2 substrate. CDK2/cyclin A2 kinase assay directed against GST-tagged fusion peptides of HEI10 Ser244 (lane 3) or Ser257 (lane 5) or their serine-alanine point mutants (lanes 4 and 6, respectively). Histone H1 is shown as a positive control to indicate kinase activity of CDK2/cyclin A2 (lane 1) whereas GST tag without fusion peptide is shown in lane 2 as a negative control. Panel A shows the Coomassie staining of radiolabelled proteins to indicate equal loading in each lane. Also shown is the detection of recombinant GST-HEI10 protein using anti-HEI10 antibodies (lane 8). Panel B shows the phospho-image of each peptide (lanes 1–6) and full length HEI10 protein (lane 7) and represents the amount of radiolabelling occurring following phosphorylation by CDK2/cyclin A2.

We considered that other possible substrates might be phosphorylated by CDK2 to potentiate its role in meiotic CO maturation. One potential substrate of CDK2 which might facilitate crossing over is HEI10 [42, 43]. We performed western blot analysis to probe for HEI10 and found that HEI10 is expressed at much higher levels in either *Cdk2KO* or *Cdk2*^*T160A*^ animals compared to WT (Fig 3, third panel from top, compare lanes 4–9 to 1–3). This is an unexpected finding but could be due most likely to compensation. It has previously been postulated that the C-terminus of HEI10, which in mouse contains two potential ‘SP’ sites Ser242 and Ser257, might represent targets of CDK phosphorylation [44]. In humans an additional ‘ideal’ CDK consensus site can be found at Thr221 but this is not conserved in other mammals. To test this hypothesis, we cloned the 15 amino acids corresponding to either Ser242 [S*PTA] or Ser257 [S*PSH] of HEI10 downstream of a glutathione-S-transferase (GST tag) and purified it from bacteria. We then used HEI10 as a substrate in an *in vitro* kinase assay with CDK2/cyclin A2 complexes. Although the common CDK-substrate, histone H1, was phosphorylated well (Fig 6A and 6B, lane 1) in this reaction, we were unable to observe incorporation of radiolabelled phosphate into HEI10 following kinase assays using CDK2/cyclin A2 (Fig 6A and 6B, lanes 2–6). This suggested that HEI10 may not be a substrate for CDK2/cyclin A2 complexes *in vitro*, since this kinase has been shown to phosphorylate many other substrates successfully (see also Fig 1). In total, we tested 4 different constructs none of which were phosphorylated by CDK2/cyclin A2 (Fig 6). Since peptides (even when fused to GST) are not always optimal substrates for kinases, we cloned full length HEI10 and purified it from bacteria. We were able to confirm proper expression of GST-tagged HEI10 by both Coomassie staining and western blotting (Fig 6A, lane 7 and 8). Nevertheless, full-length HEI10 was not phosphorylated by CDK2 (Fig 6B, lane 7). This is in contrast to the previous reports that HEI10 could be phosphorylated *in vitro* by purified CDK1/cyclin B [42]. Our results indicate that under the conditions that we used, HEI10 does not seem to be an *in vitro* substrate of CDK2/cyclin A2, a kinase with high activity. Nevertheless, we cannot conclude from this that CDK2 is not phosphorylating HEI10 *in vivo* since there are caveats that need to be considered. These include that (i) recombinant HEI10 (both peptides and full-length) may not be folded properly, (ii) cyclin A2 is not the correct partner of CDK2 in meiosis [CDK2 is interacting with other cyclin-like partners like Speedy proteins, etc.], or (iii) that other co-factors are needed in order for HEI10 to become phosphorylated.

## Discussion

CDK2 has been studied extensively and has functions in the mitotic cell cycle and during meiosis. Nevertheless, only a fraction of the CDK2 substrates are known, which limits our understanding of this important kinase. Since CDK2 plays important roles in fertility and has been implicated to affect prophase of meiosis I on multiple levels [70], our aim was to investigate some of the potential substrates that may be responsible for the functions of CDK2 in meiosis. Although some CDK2 substrates are known [47, 48], they were identified in cell lines during the mitotic cell cycle. Therefore, our knowledge of meiotic CDK2 substrates is rudimentary. Here we identify PMS2, PMS1, and most likely MLH1 as potential CDK2 substrates *in vitro*. One interesting aspect is that the stability of PMS2 has been reported to be dependent on MLH1 [61–63]. Cell lines that do not express MLH1, express very low levels of the PMS2 protein despite that *Pms2* mRNA is expressed at normal levels [61]. Although this is compelling evidence from mitotically dividing cells, the situation in meiosis may not be the same. For example, the meiotic arrest phenotype of knockout mice for *Pms2* (defect in synapsis) and *Mlh1* (post synapsis defect) is different. In addition, MLH1 is not expressed in early prophase I and therefore it would be difficult to explain the early functions of PMS2 (one would expect the PMS2 protein to be unstable under these conditions). In summary, we may not be able to extrapolate all the data from mitotically dividing cells to meiosis and there is a possibility that the stability of PMS2 is not only regulated by MLH1 but possibly also by other factors. Our own results indicate that the PMS2 protein levels are slightly increased in *Cdk2KO* and *Cdk2*^*T160A*^ mice (Fig 3). Although we have not been able to detect the MLH1 protein directly, using MSH2 as a proxy, it is possible MLH1 is not expressed in these mutant mice. If MSH2 expression serves as a reliable proxy for MLH1 expression, we have to wonder how PMS2 can be stable in testis extracts of *Cdk2KO* and *Cdk2*^*T160A*^ mice? There is a clear need to study the stability of PMS2 during meiosis in more details in the future.

**Fig 7:**
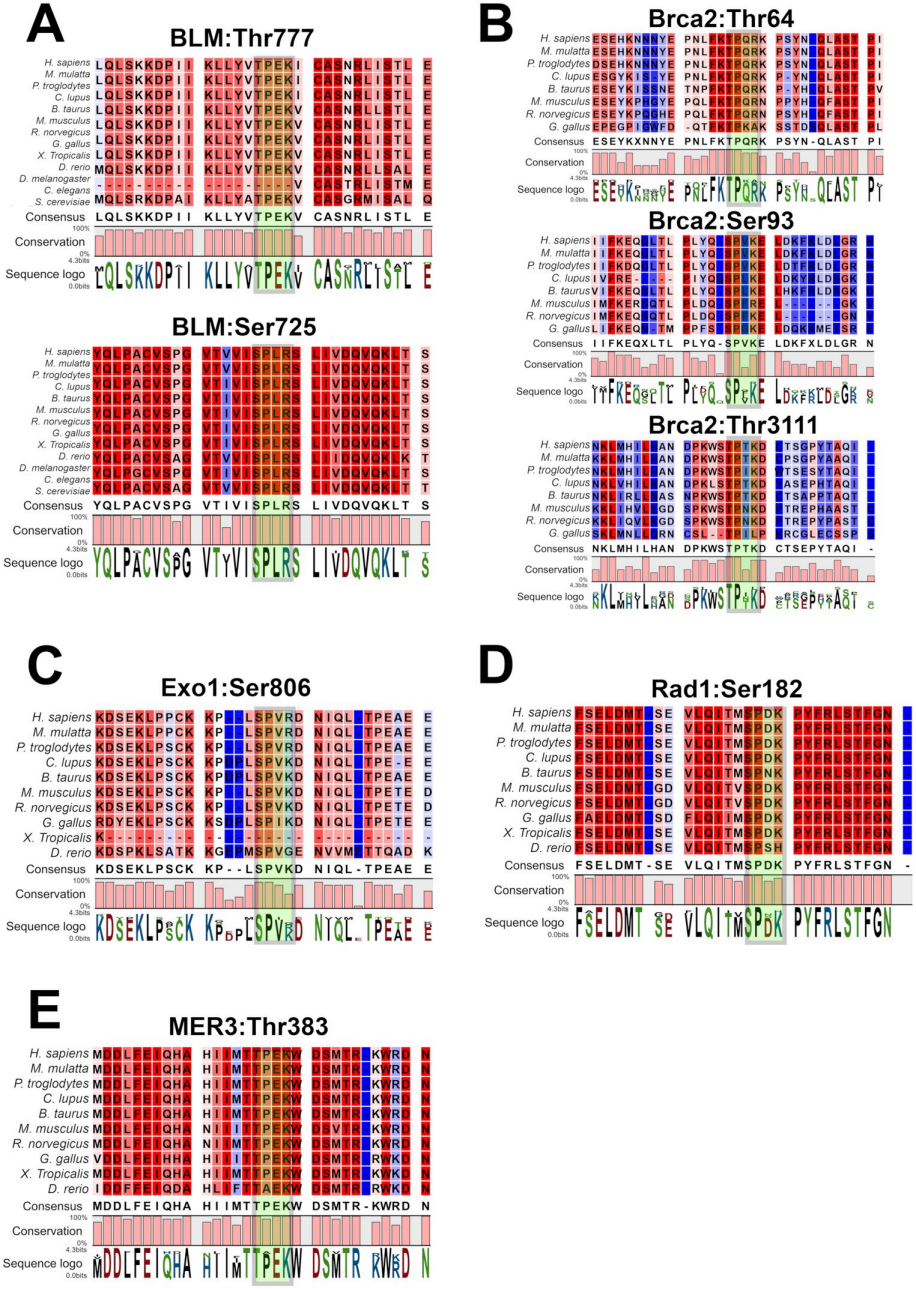
Other potential meiotic substrates of CDK2. Sequence alignment of the evolutionary conservation of various potential CDK consensus sites in meiotic proteins: BLM Thr777/Ser725 (A), BRCA2 Thr64/Ser93/Thr3111 (B), Exo1 Ser806 (C), RAD1 Ser182 (D), and MER3 Thr383 (E). Amino acid positions are labelled relative to the transcripts from *Mus musculus*. The CDK consensus site in each sequence is highlighted by a green box.

Under the conditions that we have used, we find that CDK2/cyclin A2, which we have used for all the experiments, does not phosphorylate HEI10. Although this should not serve as a final conclusion, it does indicate that if HEI10 is a CDK2 substrate *in vivo*, it will require specific conditions or cofactors. In addition to HEI10, several other proteins are known to localise to late recombination nodules with similar timing to CDK2 or are known to affect the meiotic recombination process in a way which might disrupt CO formation. We searched the amino acid sequences of these proteins for CDK consensus sites for phosphorylation to determine whether they might be potential CDK substrates. Mouse RNF212 has 10 SP/TP sites but none of these were optimal CDK sites and none of these were evolutionarily conserved. Several meiotic proteins have well conserved CDK consensus sites. These include Ser725 and Thr777 of the BLM helicase, Ser806 of EXO1, Ser93, Thr64 and Thr3111 of BRCA2, Ser182 of RAD1, and Thr384 of MER3 (Fig 7).

BLM is thought to be involved in CO suppression by competing with the CO machinery to convert meiotic recombination intermediates into NCOs [71]. BLM has been reported to be phosphorylated by CDK2 but at the N-terminus [72]. RAD1 is part of the 9-1-1 DNA clamp complex [73]. In yeast the 9-1-1 complex is needed for the loading of the ZMM (pro-CO) factors at meiotic recombination sites [74]. BRCA2 is thought to be involved in the second end capture process which leads to the conversion of a DNA intermediate into a double Holliday junction [75] and has been reported to be phosphorylated at the C-terminus [76]. Murine MER3 is known to be directly involved in CO formation [13], a function conserved in other eukaryotes [77, 78]. Unlike the *Cdk2*^*T160A*^ mutant, however, *Mer3* mutants display synapsis defects [13]. Probably the most similar phenotype to the *Cdk2*^*T160A*^ mutant is the *Exo1* knockout mouse model. EXO1 is thought to be part of two different events in meiosis. The first one is an early step of meiotic recombination, end resection of meiotic DSBs and the second one is a later function in CO formation [46]. The functions of EXO1 in meiosis is independent of its exonuclease activity and is suggested to be due to a scaffolding function of EXO1 which potentiates CO formation [12].

## Conclusions

We have analysed potential meiotic kinase substrates and provide evidence that PMS2 (and PMS1) and to some degree MLH1 are phosphorylated *in vitro* by CDK2. In addition, BLM, BRCA2, EXO1, RAD1, and MER3 contain consensus CDK phosphorylation sites and therefore are additional potential CDK2 substrates that could be tested in the future. The limitations of our study, which have been mentioned in the text, are that our approach was *in silico* and *in vitro*. Therefore, we are unable to provide evidence that PMS2, PMS1, or MLH1 are phosphorylated by CDK2 during meiosis *in vivo*. To obtain an *in vivo* proof that a substrate is phosphorylated by CDK2 is far from trivial since even generating a mouse carrying the *Pms2*^*T337A*^ mutation would not prove that this site is phosphorylated by CDK2 but at least would indicate that the posttranslational modification of PMS2 at Thr337 is important for meiotic progression. There are additional limitations of our study which relate to the cyclin partner of CDK2. In our study, we used cyclin A2 to activate CDK2 since our past experience with CDK2/cyclin A2 complexes have shown that these are highly active (which is also shown in Fig 6B, lane 1). Nevertheless, CDK2 may partner with other types of cyclins during meiosis including cyclin E, Speedy proteins [79], CNTD1 [11], or PRR19 [80]. These cyclins/cyclin-like proteins may potentially affect the substrate specificity of CDK2 complexes and therefore would affect the outcome of our experiments. Finally, we cannot exclude that other (adapter)proteins or the environment could affect the meiotic substrate specificity of CDK2, which are not accounted for in our *in vitro* experiments. Despite all these caveats and limitations, we believe that our results are interesting and could stimulate researchers to analyze further CDK2 substrates during meiosis.

In conclusion, we have identified potential substrates of CDK2 with functions in meiosis. These results will need to be verified by *in vivo* experiments, which will require new methodology or a tour-de-force approach. Our data also suggests that the stability of PMS2-MLH1 in meiosis may be regulated in a different way than in mitotically dividing cells.

## Supporting information

S1 Raw images (PDF)
