# Supplementary material for: Identification PMS1 and PMS2 as potential meiotic substrates of CDK2 activity
Source: PLoS One. 2023 Mar 23;18(3):e0283590. doi: 10.1371/journal.pone.0283590 (PMC10035876; doi:10.1371/journal.pone.0283590)

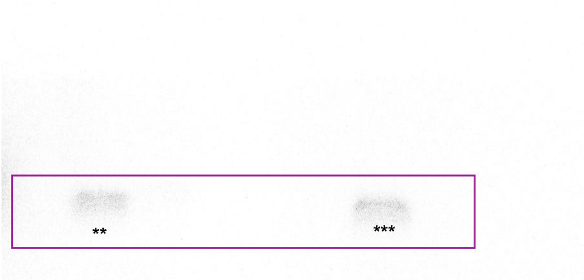

** PMS1 T311 peptide
***PMS2 T337 peptide

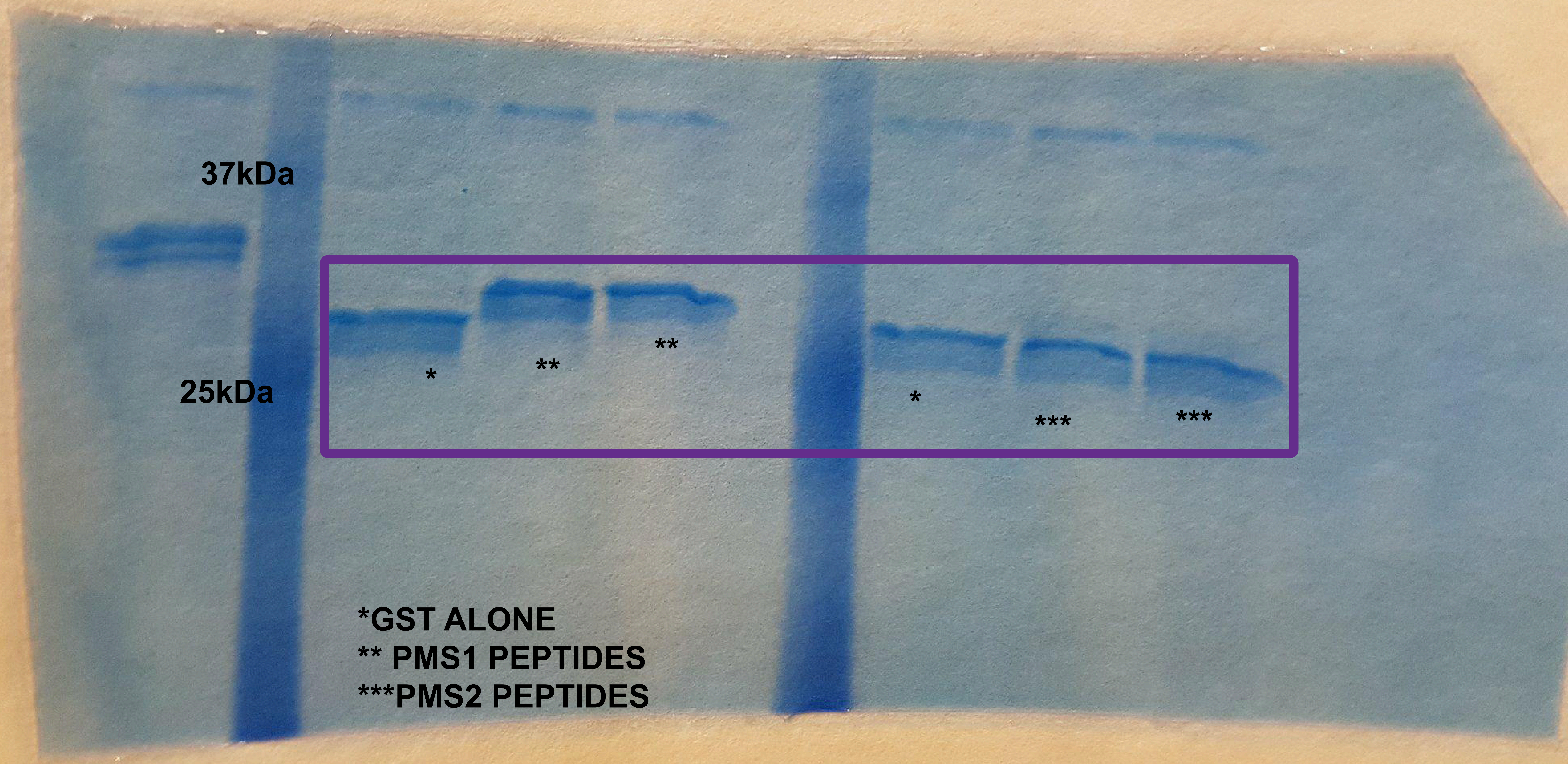
37kDa
25kDa
*
**
**
*
***
***
*GST ALONE
** PMS1 PEPTIDES
***PMS2 PEPTIDES

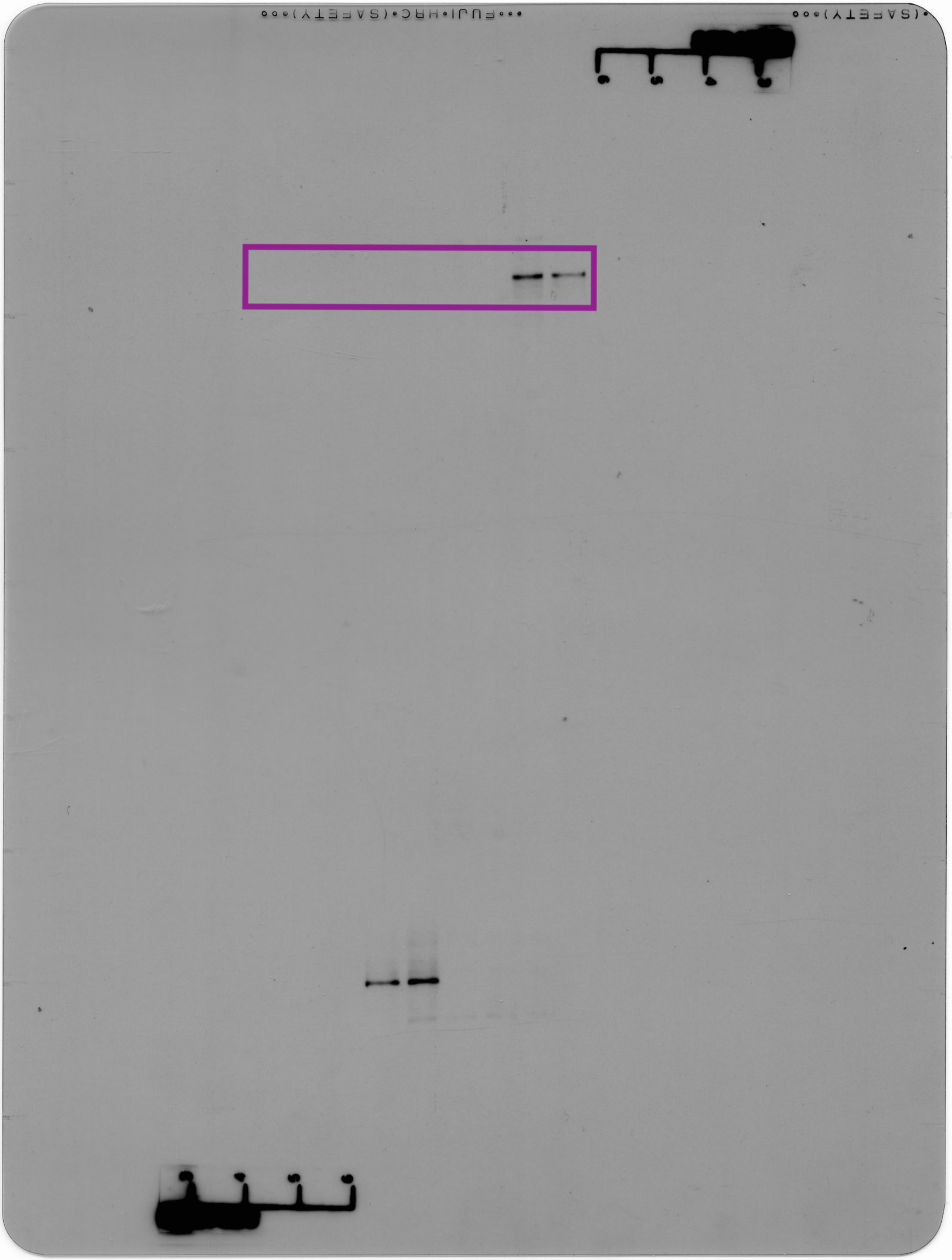
FUJI • HRC • (SAFETY)

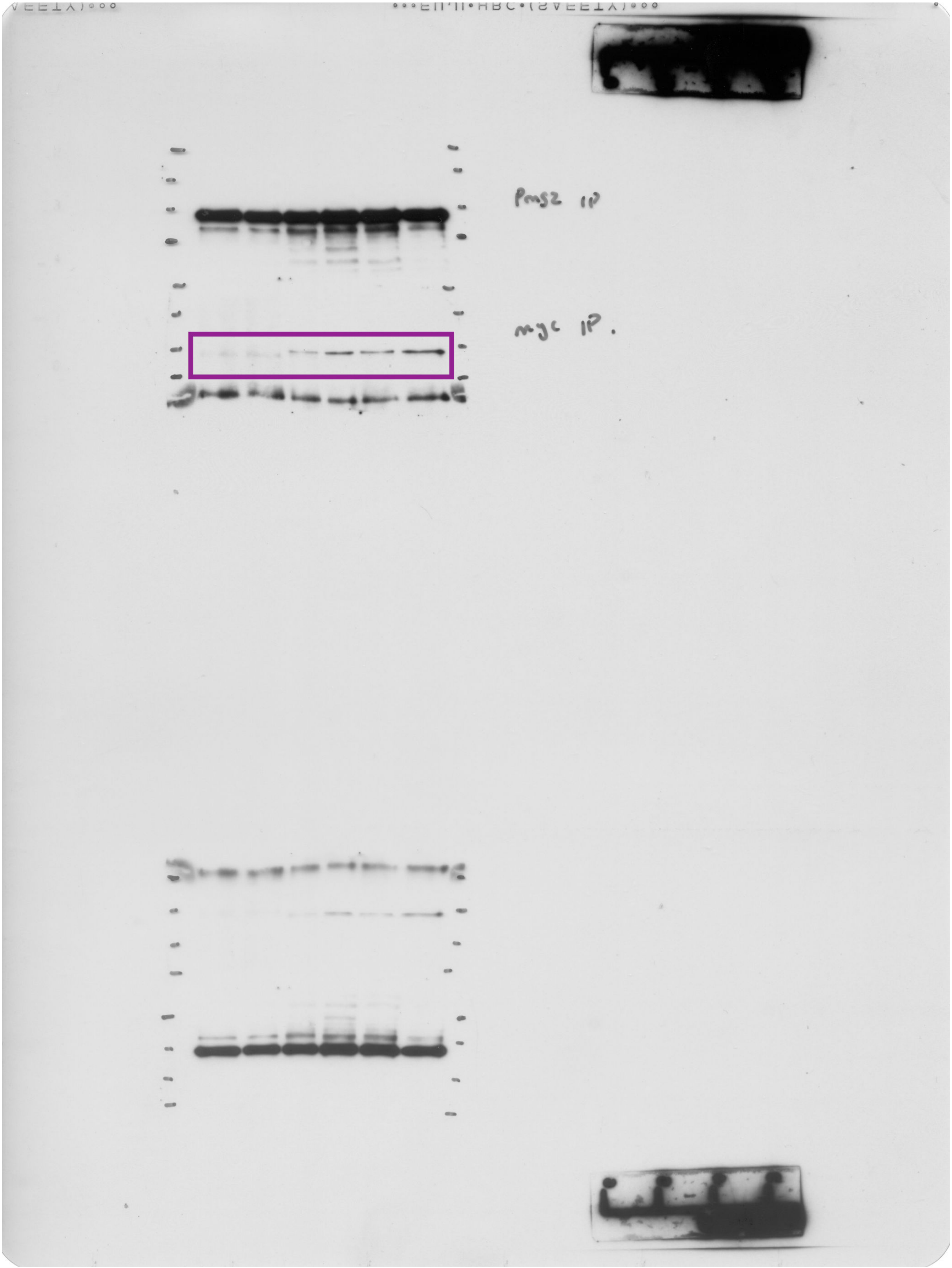
Pms2 IP
myc IP.

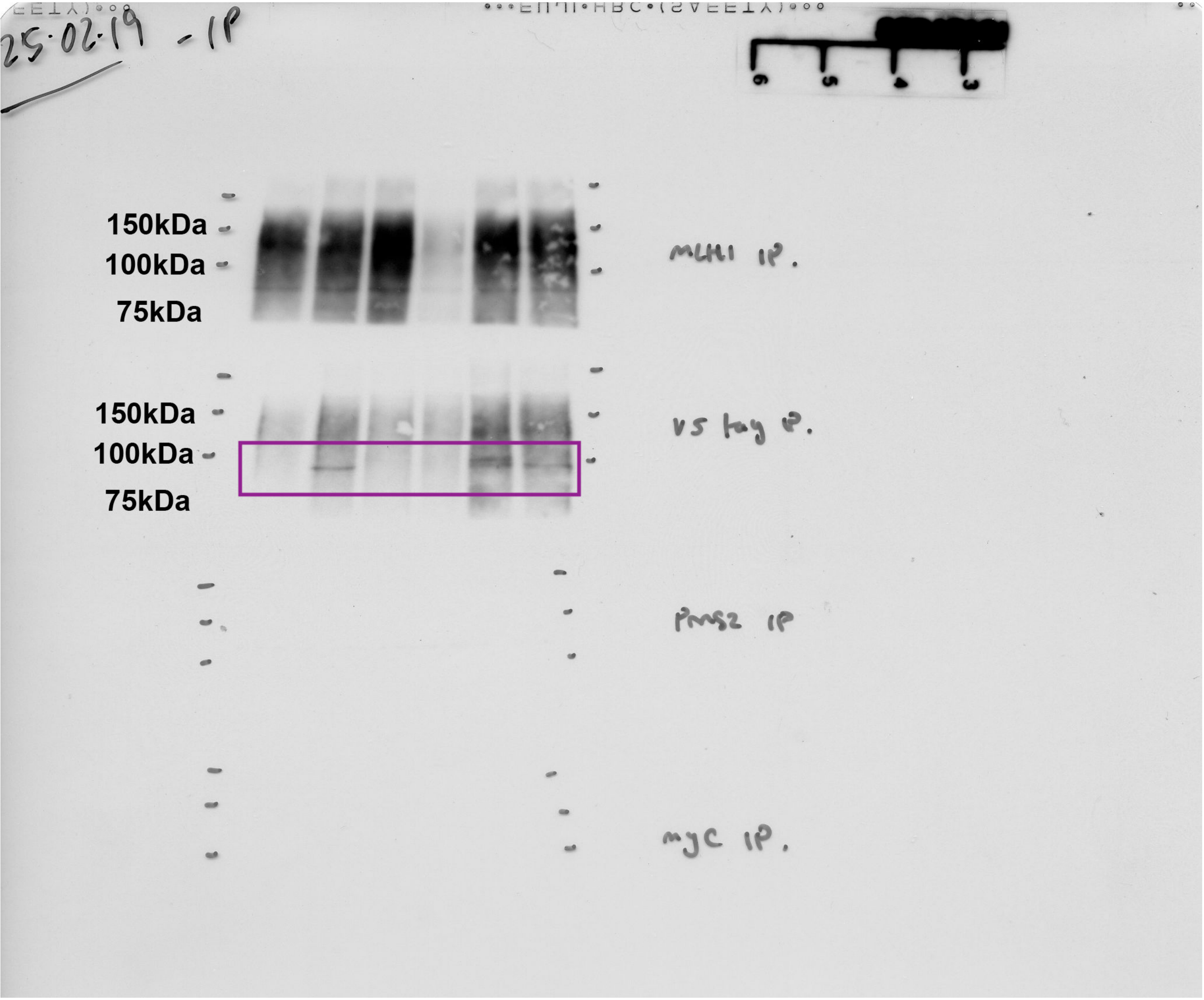

25.02.19 - IP
150kDa
100kDa
75kDa
MLH1 IP.
150kDa
100kDa
75kDa
V5 tag IP.
PMS2 IP
myc IP.

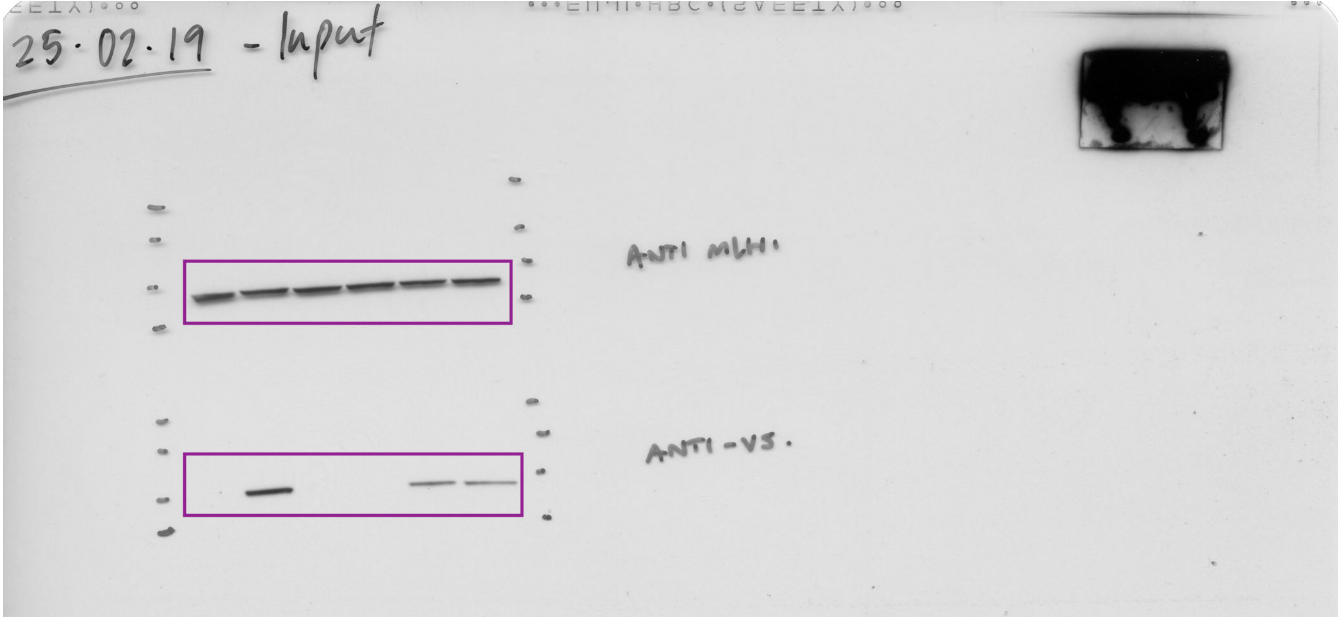
25·02·19 - Input
ANTI MLH1
ANTI-V5.

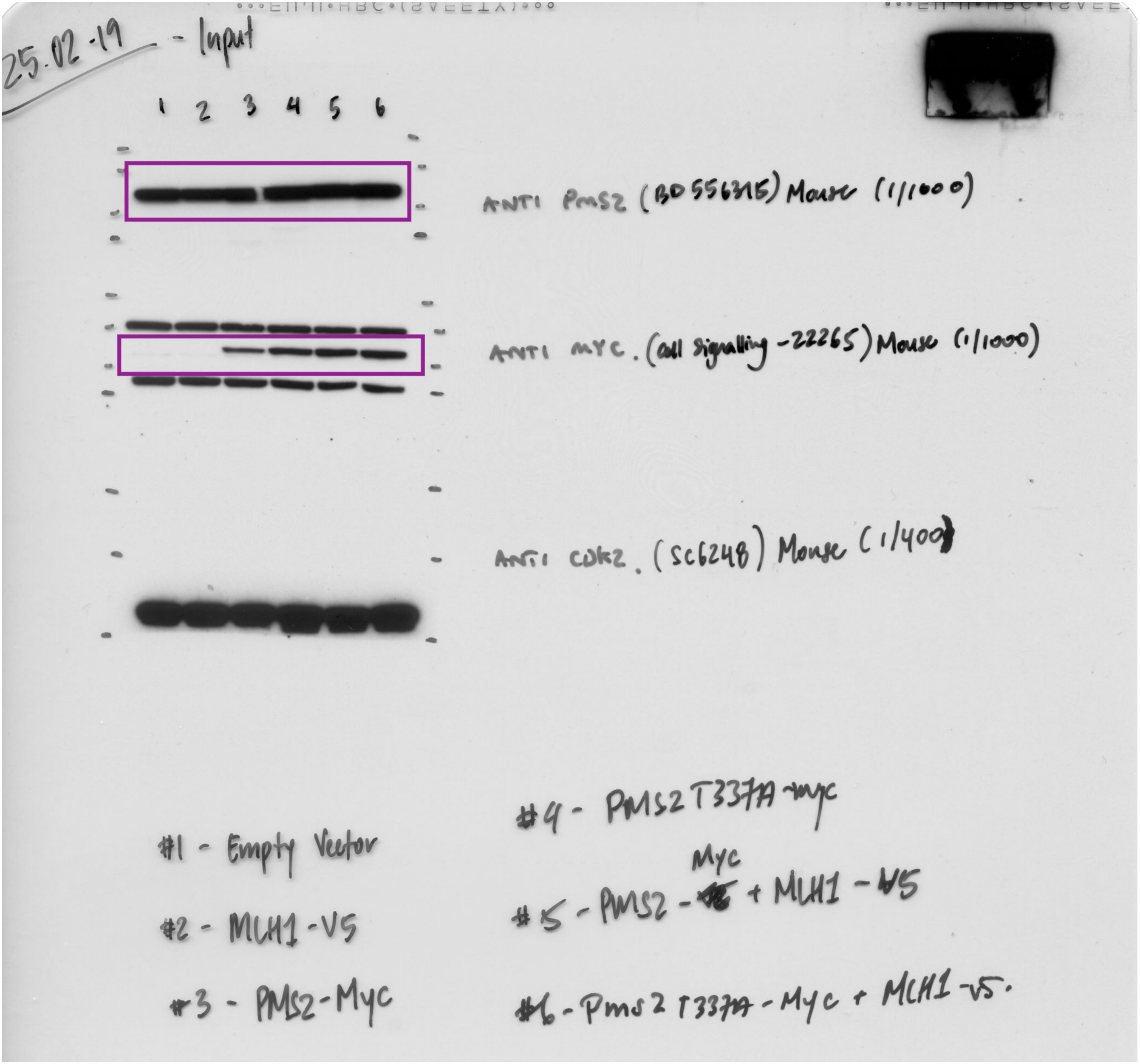
25.02.19 - Input
1 2 3 4 5 6
ANTI PmS2 (BD 556315) Mouse (1/1000)
ANTI MYC. (cell signalling -22265) Mouse (1/1000)
ANTI CDK2. (SC6248) Mouse (1/400)
#1 - Empty Vector
#2 - MLH1 - V5
#3 - PMS2-Myc
#4 - PMS2 T337A-myc
#5 - PMS2 - Myc + MLH1 - V5
#6 - Pms2 T337A - Myc + MLH1 -V5.

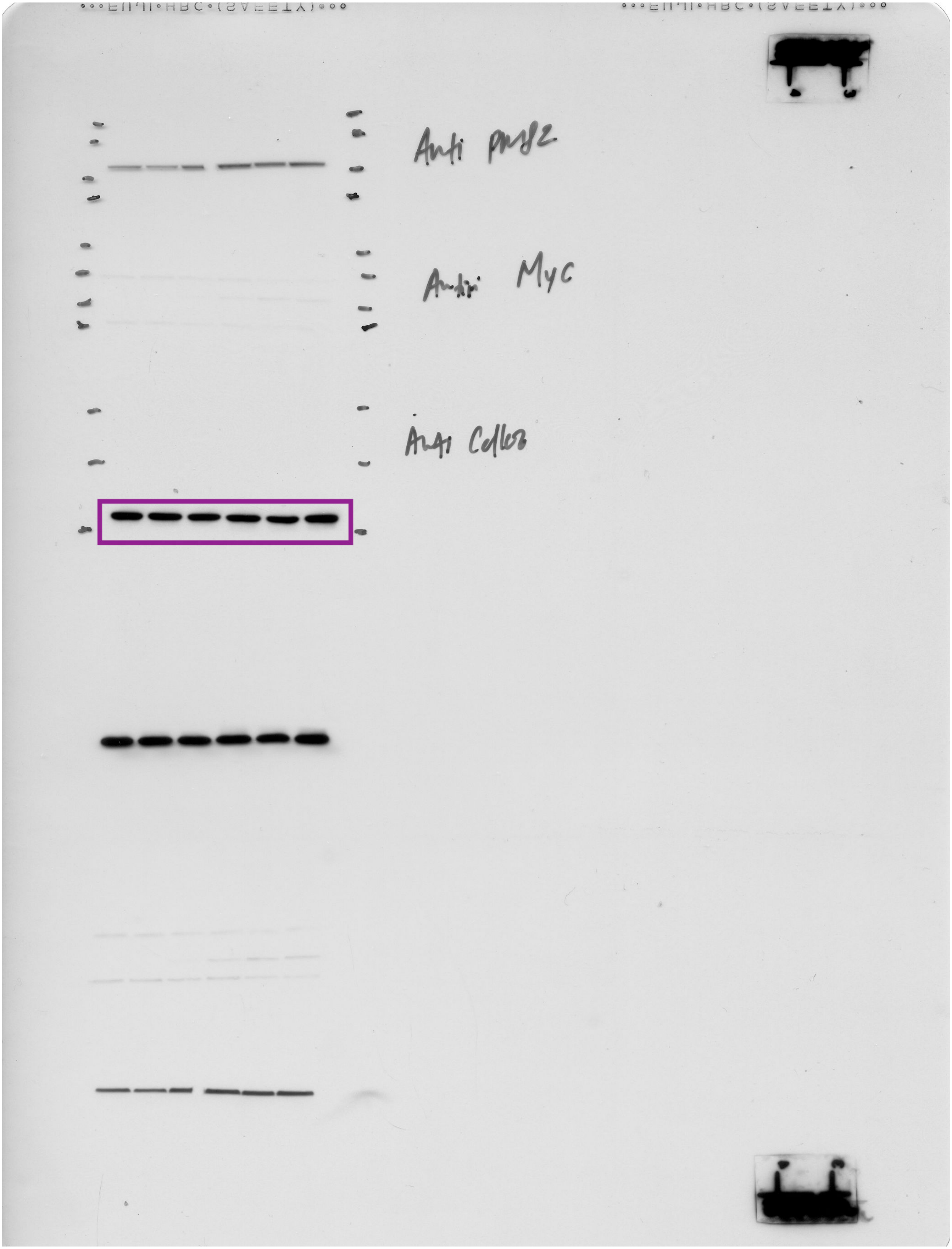
Anti Myc

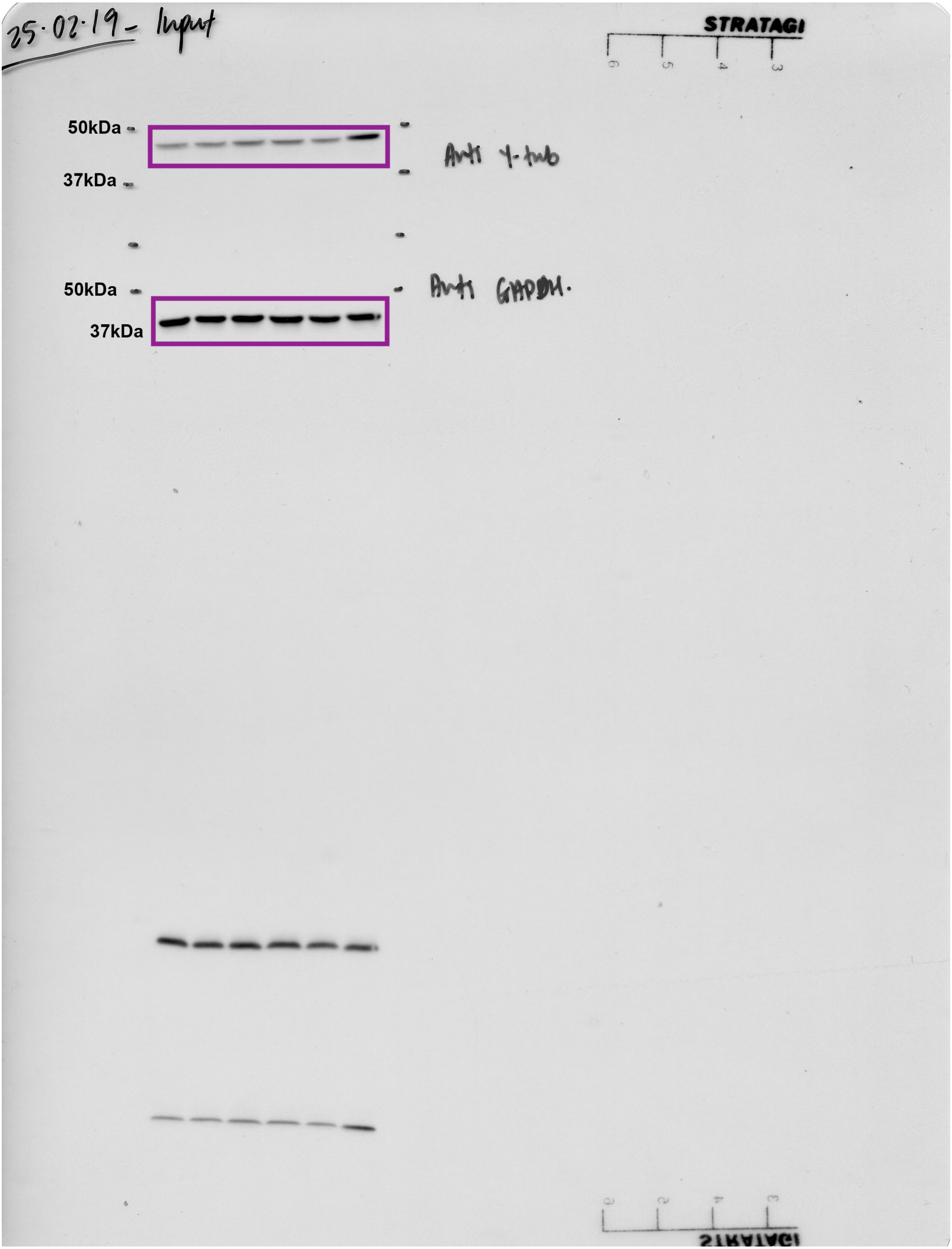
25·02·19 - Input
STRATAGENE
50kDa
37kDa
Anti γ-tub
50kDa
37kDa
Anti GAPDH.

PMS2/PMS2T337A Kinase assay Fig2C

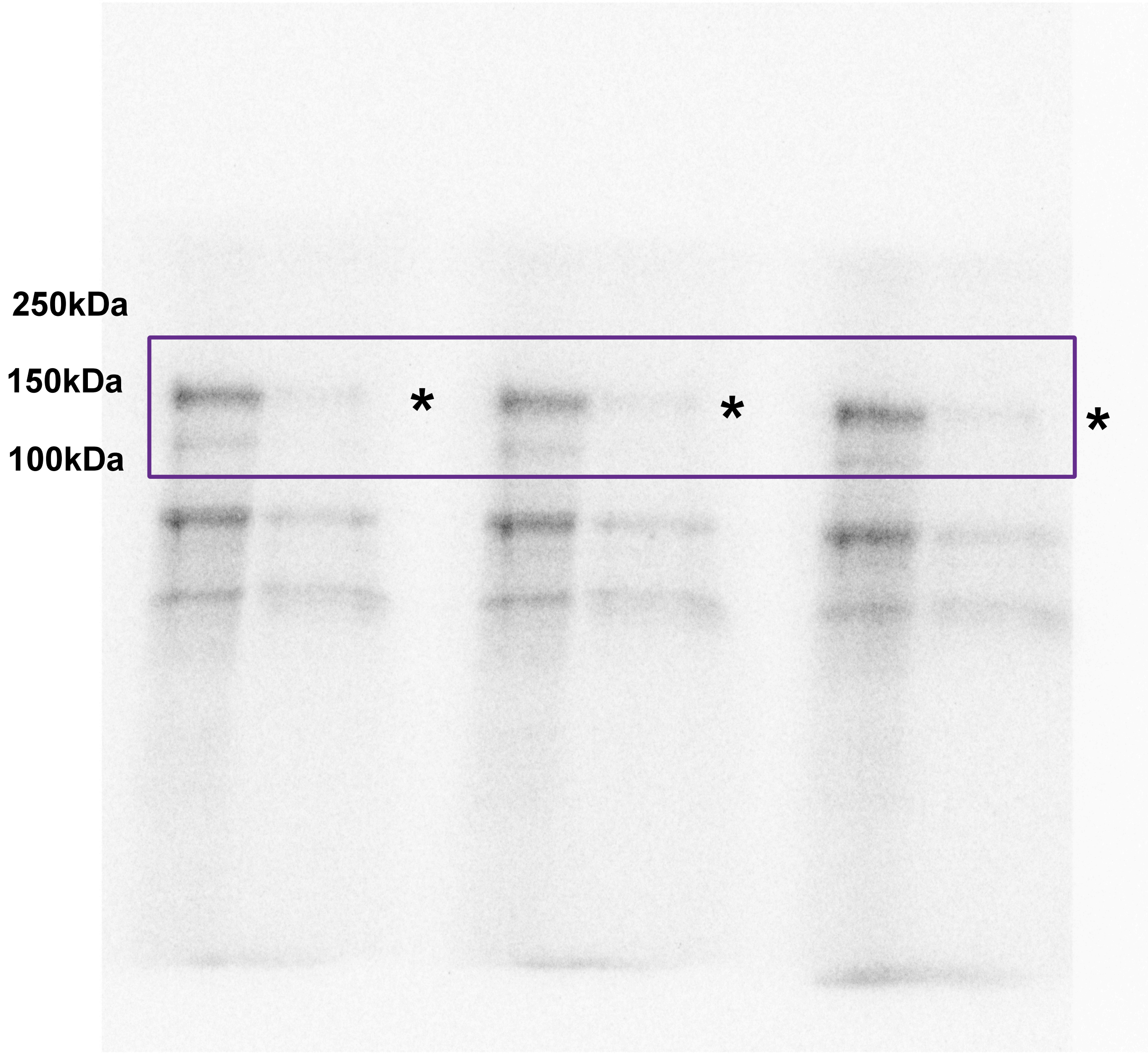

*PMS2/PMS2^{T337A}

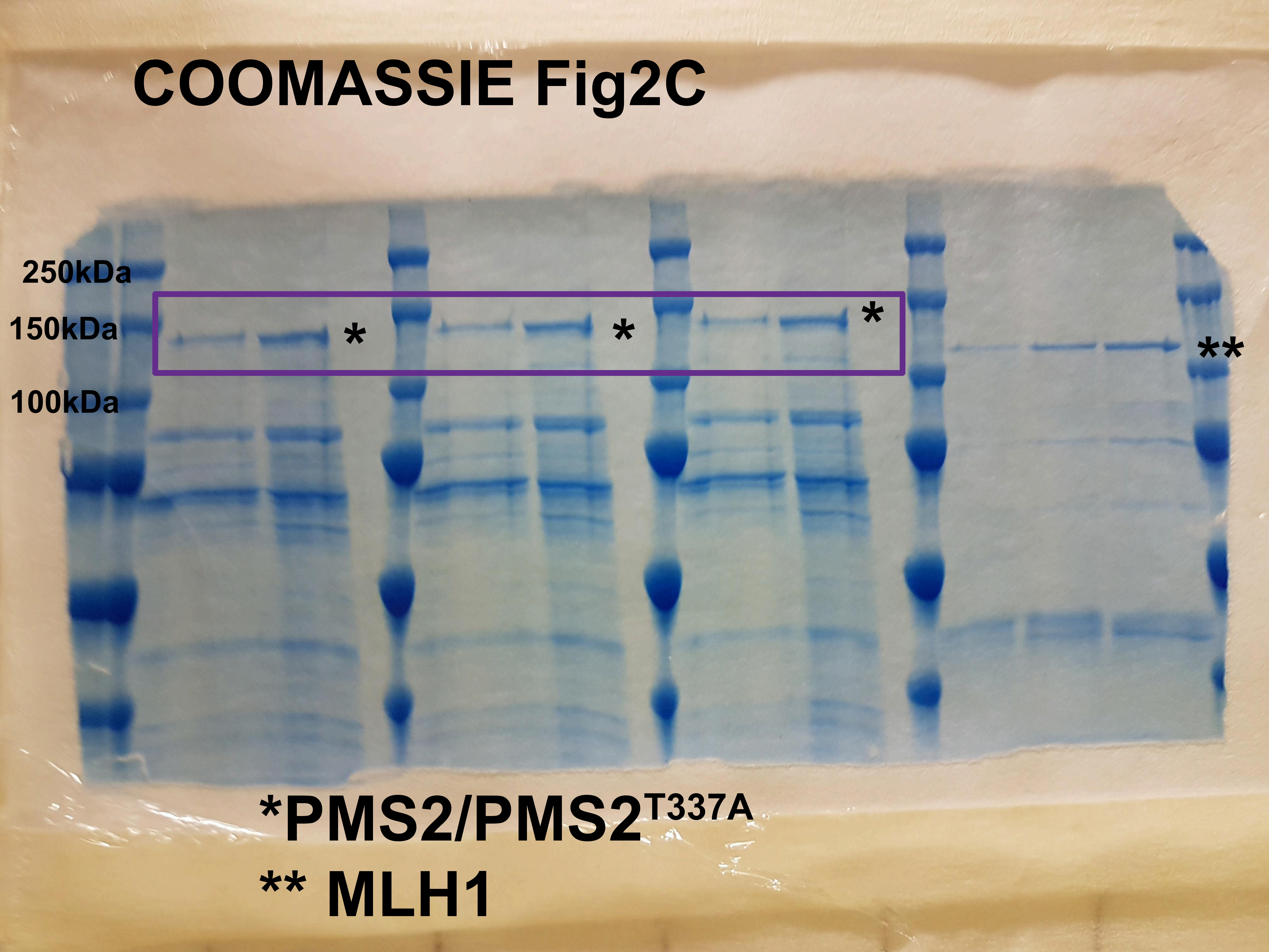
COOMASSIE Fig2C
250kDa
150kDa
100kDa
*
*
*
**
*PMS2/PMS2T337A
** MLH1

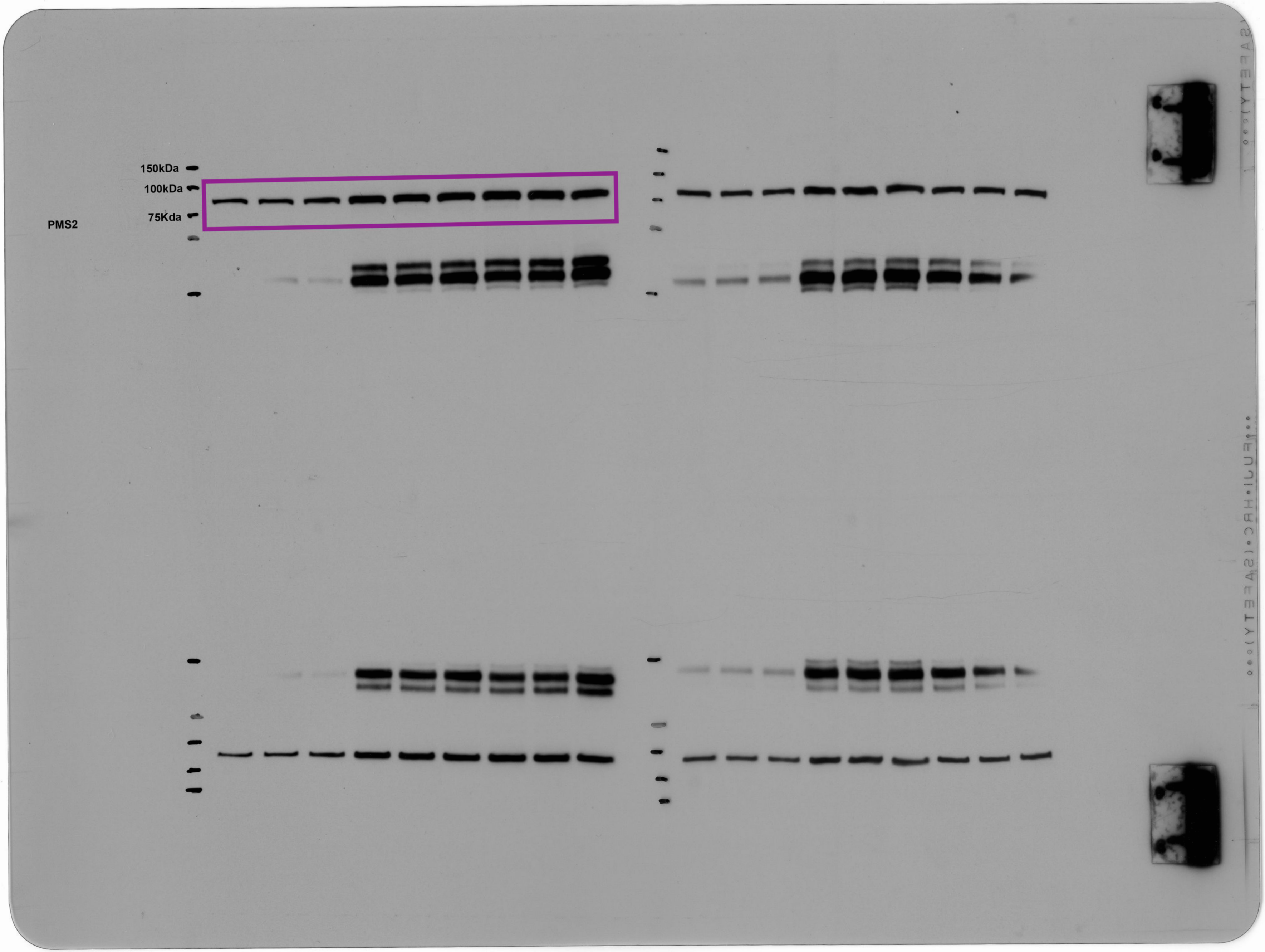
150kDa
100kDa
75Kda
PMS2

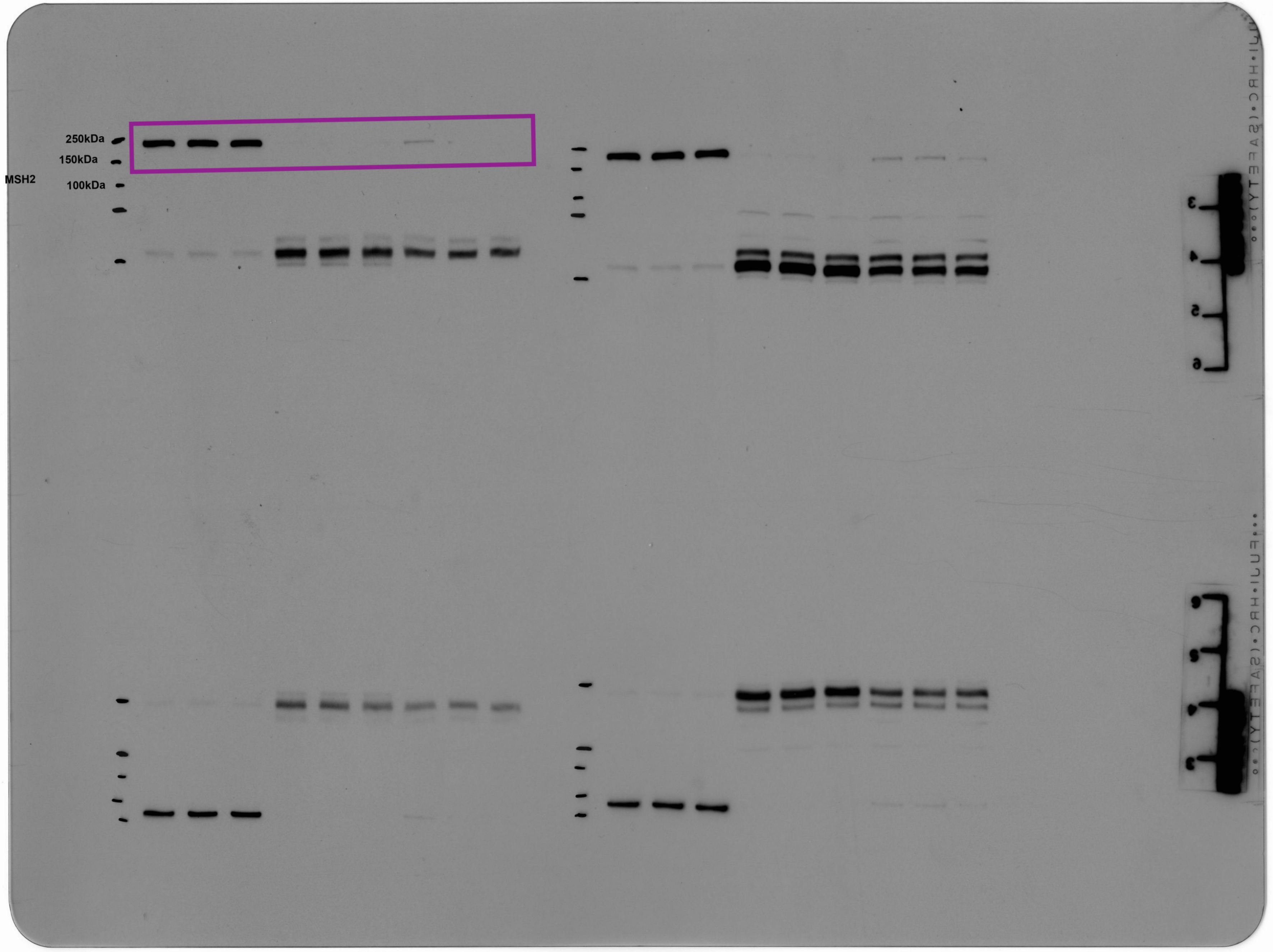
250kDa
150kDa
MSH2
100kDa

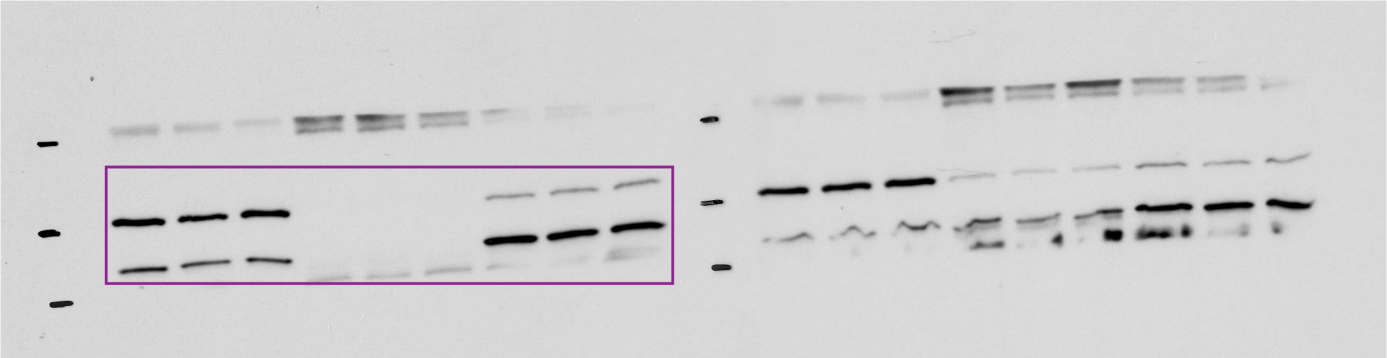

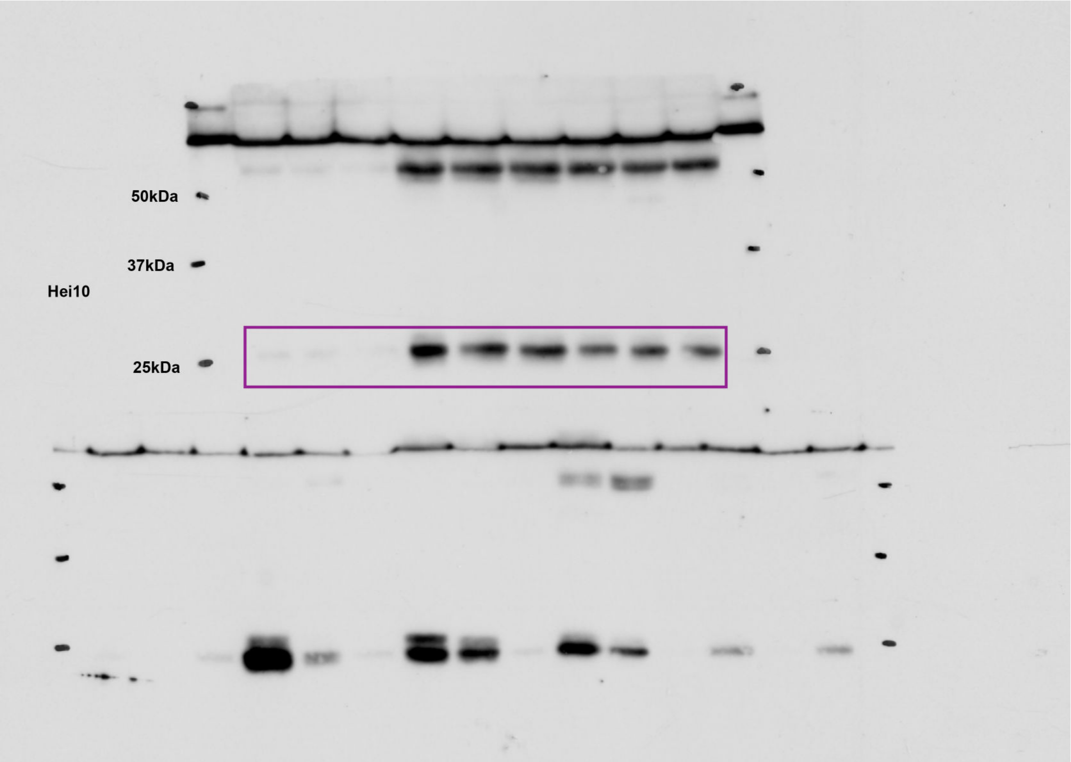
50kDa
37kDa
Hei10
25kDa

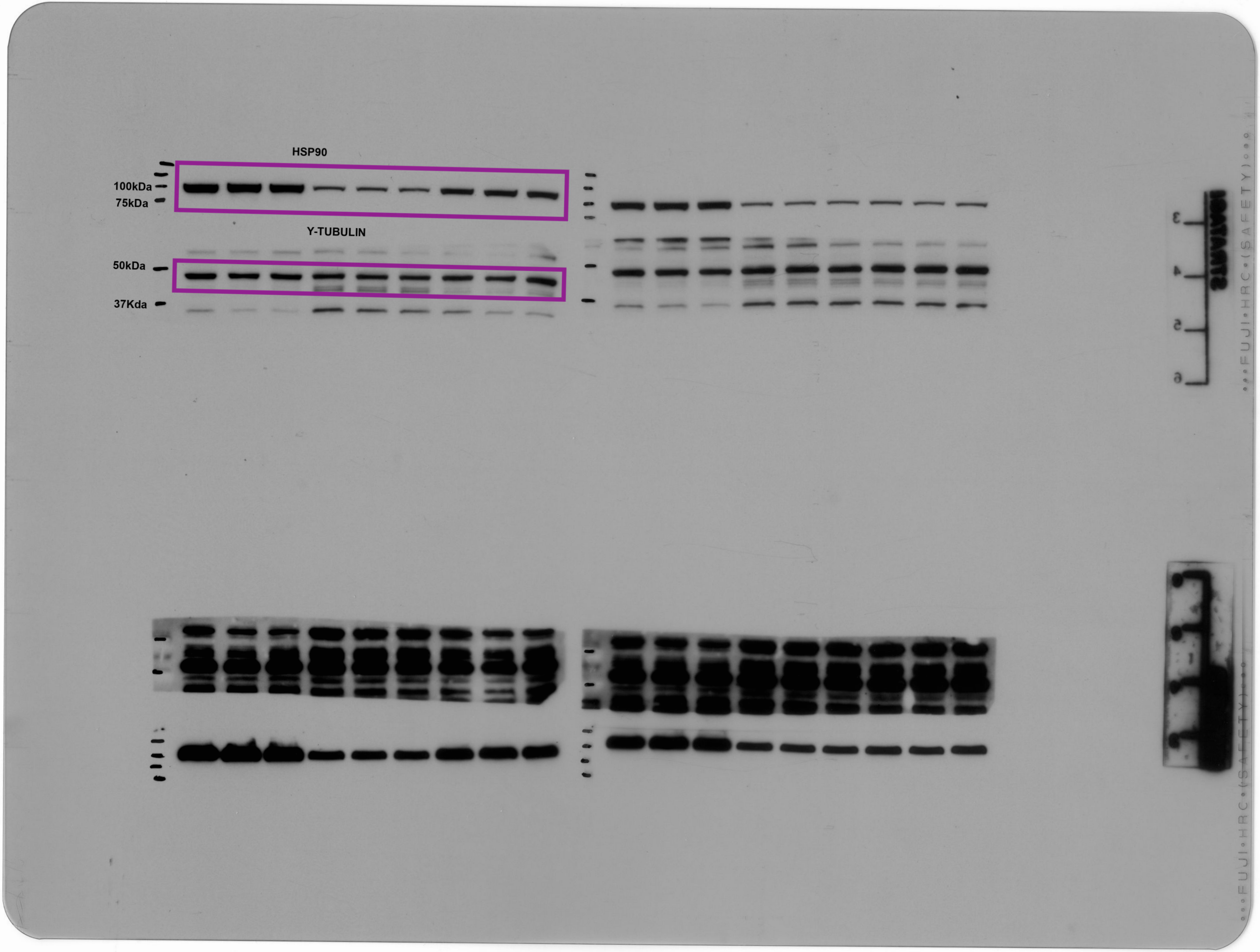
HSP90
100kDa
75kDa
Y-TUBULIN
50kDa
37Kda

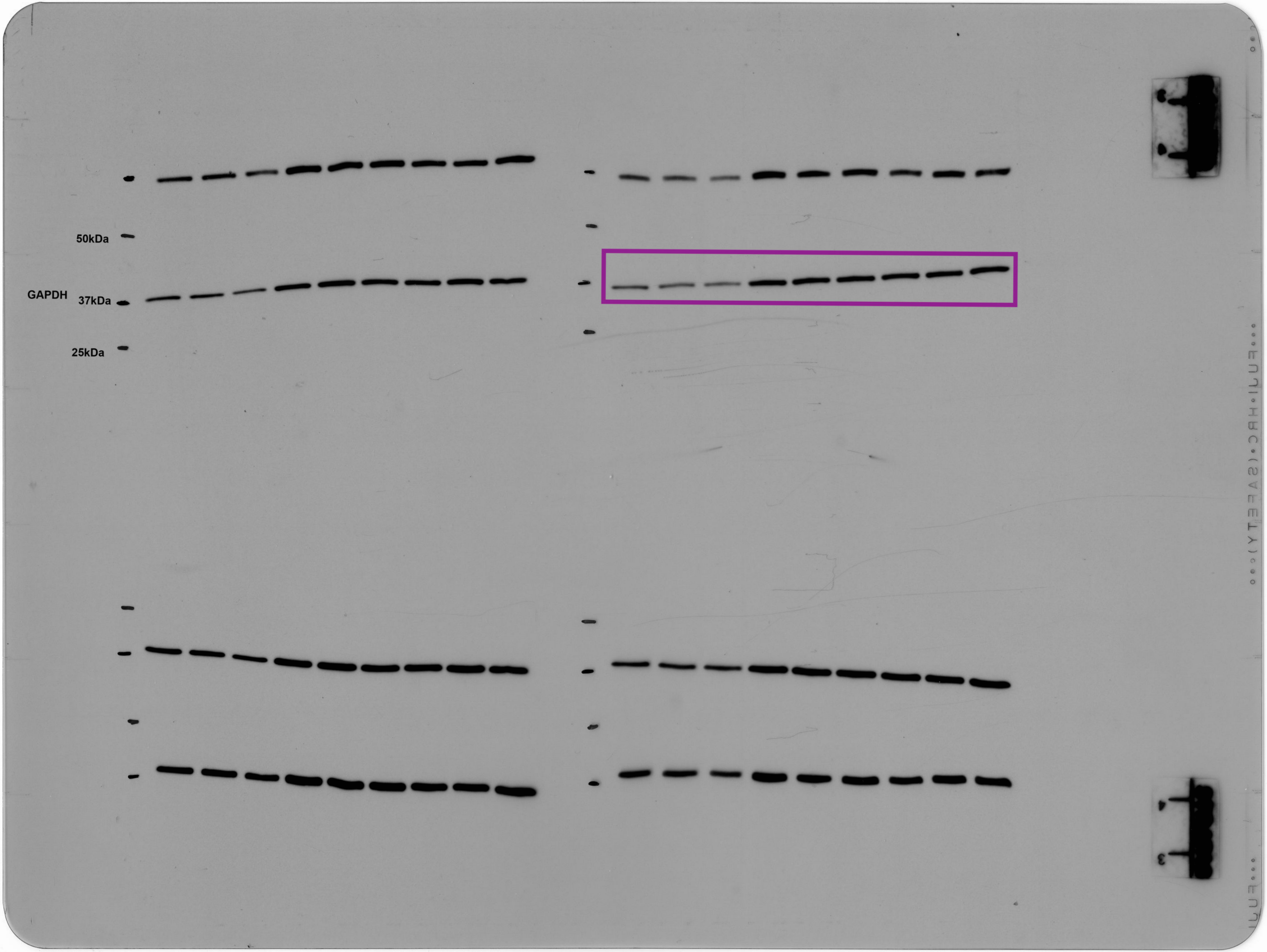
50kDa
GAPDH
37kDa
25kDa

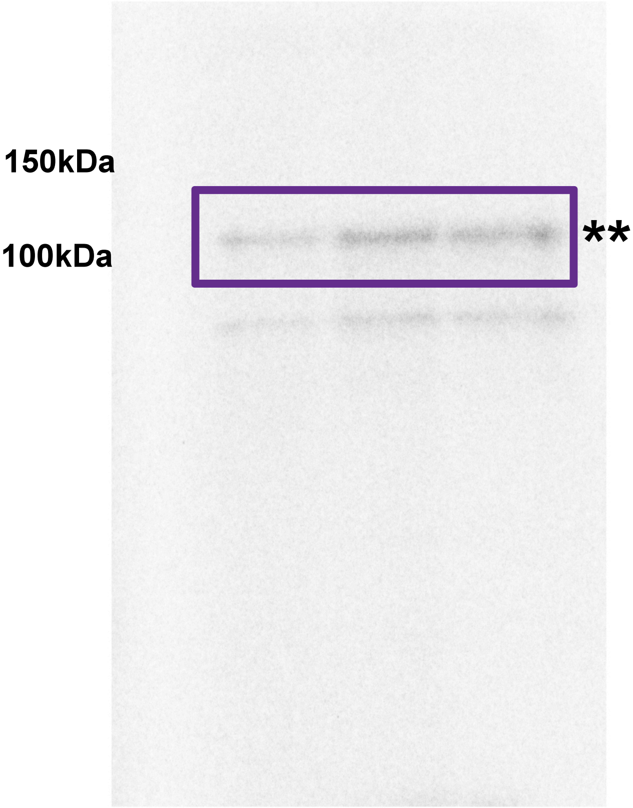

** MLH1

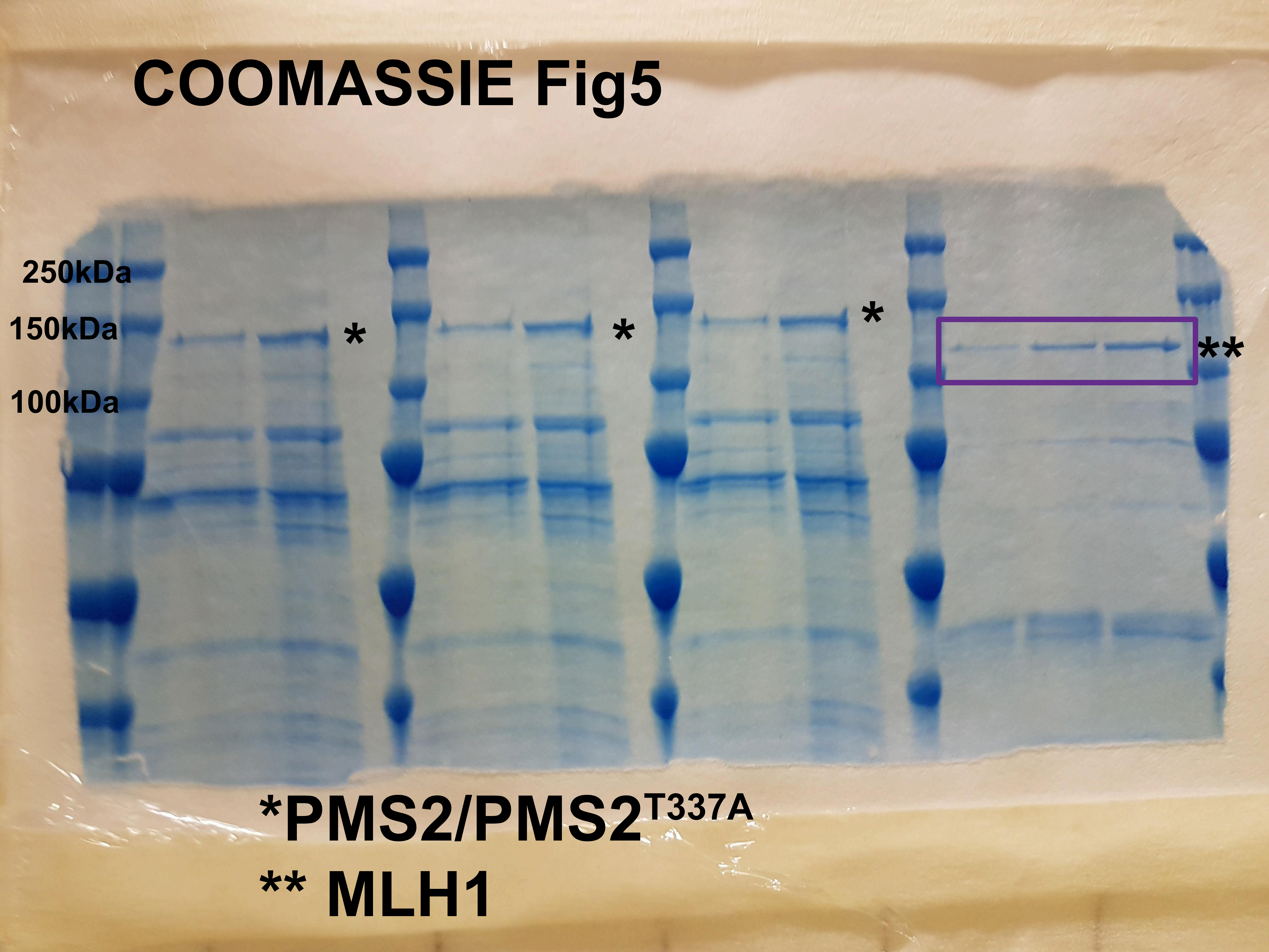
COOMASSIE Fig5
250kDa
150kDa
100kDa
*
*
*
**
*PMS2/PMS2T337A
** MLH1

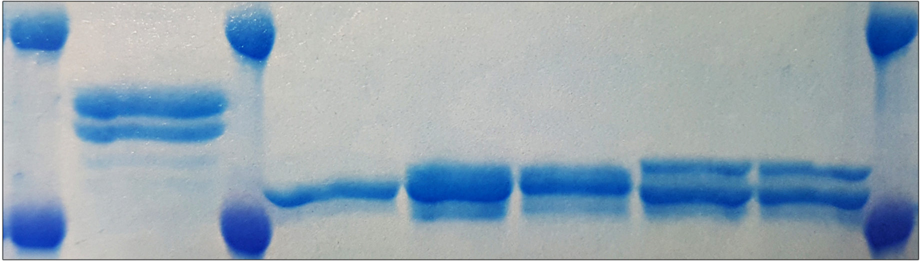

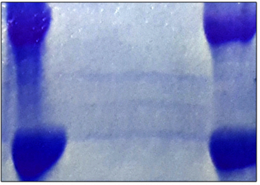

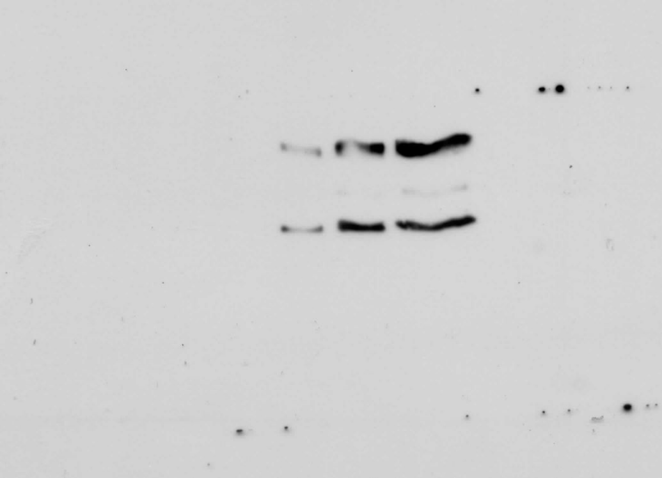

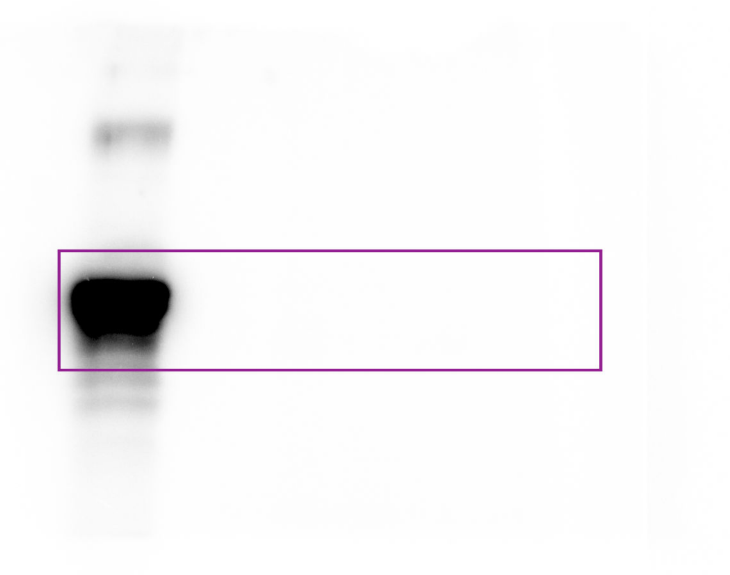

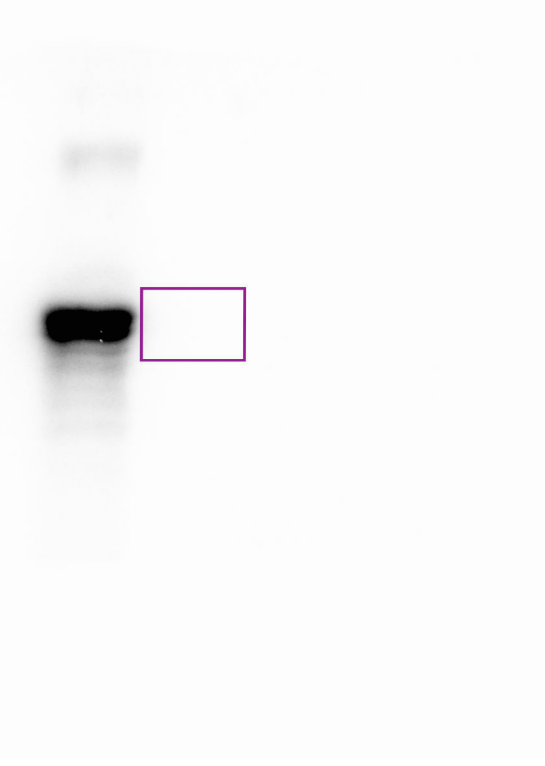

Supplement: S1 Raw images — (PDF) [file pone.0283590.s001.pdf]
